# Identification of PKS-NRPS Hybrid Metabolites in Marine-Derived *Penicillium oxalicum*

**DOI:** 10.3390/md20080523

**Published:** 2022-08-16

**Authors:** Hongcheng Li, Wei Zhang, Xuan Zhang, Shen Tang, Ping Men, Mengyi Xiong, Zhimin Li, Yongyu Zhang, Xuenian Huang, Xuefeng Lu

**Affiliations:** 1 Shandong Provincial Key Laboratory of Synthetic Biology, Qingdao Institute of Bioenergy and Bioprocess Technology, Chinese Academy of Sciences, Qingdao 266101, China; 2 School of Medicine and Pharmacy, Ocean University of China, Qingdao 266003, China; 3 Jiangxi Engineering Laboratory for the Development and Utilization of Agricultural Microbial Resources, College of Bioscience and Bioengineering, Jiangxi Agricultural University, Nanchang 330045, China; 4 Shandong Energy Institute, Qingdao 266101, China; 5 Qingdao New Energy Shandong Laboratory, Qingdao 266101, China; 6 University of Chinese Academy of Sciences, Beijing 100049, China; 7 Marine Biology and Biotechnology Laboratory, Qingdao National Laboratory for Marine Science and Technology, Qingdao 266101, China; 8 Key Laboratory of Biofuels, Qingdao Institute of Bioenergy and Bioprocess Technology, Chinese Academy of Sciences, Qingdao 266101, China

**Keywords:** marine-derived fungi, oxopyrrolidines, gene cluster, biosynthesis

## Abstract

Filamentous fungi are abundant resources of bioactive natural products. Here, 151 marine-derived fungi were collected from the north Yellow Sea and identified by an internal transcribed spacer (ITS) sequence. The crude extracts of all strains were evaluated for their antimicrobial activities and analyzed by HPLC fingerprint. Based on these, strain *Penicillium oxalicum* MEFC104 was selected for further investigation. Two new polyketide–amino acid hybrid compounds with feature structures of tetramic acid, oxopyrrolidine A and B, were isolated. Their planner structures were assigned by HRESIMS and 1D/2D NMR experiments. The absolute configurations were determined by modified Mosher’s method, *J*-based configuration analysis, and ECD calculations. Furthermore, the biosynthetic pathway was identified by bioinformatic analysis and gene-deletion experiments. This study established a link between oxopyrrolidines and the corresponding biosynthesis genes in *P. oxalicum*.

## 1. Introduction

The pursuit of drug discovery over many decades has emphasized that fungi are versatile producers of natural products with unpredictable architectures and bioactivities. Some famous molecules produced by fungi, such as the antibiotics penicillin [1] and echinocandins [2], the cholesterol-lowering agent lovastatin [3], and immunosuppressant cyclosporine [4], have been applied to clinical trials and play essential roles in human health defense. The marine environment is characterized by high salt, high pressure, low temperature, low light, and oligotrophic conditions. Compared to the terrestrial environment, the harsh conditions in the marine environment might have enabled fungi to gain particular biosynthetic pathways of structurally unique and biologically active secondary metabolites, some of which might shed light on the further exploration of new drug leads [5].

Fungal polyketide–amino acid hybrids are a large family of secondary metabolites produced by PKS-NRPS assembly lines. Due to the common biosynthetic logic, most polyketide–amino acid hybrid molecules arise from precursors with the substructure of tetramic acid. The pyrrolidine-2,4-dione moiety of tetramic acid derivatives enables these molecules to exhibit antibiotic [6], antifungal [7], and cytotoxic activities [8]. Meanwhile, the complex structures and diverse activities of tetramic acid derivatives have inspired various studies on biosynthesis mechanisms and chemical modifications [9], which provide more clues for mining bioactive natural products.

In our continuing investigation of marine microorganisms and related natural products, we isolated 151 fungi strains from marine samples gathered around the north Yellow Sea. Based on bioactivity screening and chemical profiles, *P. oxalicum* MEFC104 was selected for further study. Two new tetramic acid derivatives, oxopyrrolidine A (**1**) and oxopyrrolidine B (**2**), were obtained. Their absolute configurations were determined by modified Mosher’s method, *J*-based configuration analysis, and ECD calculations. In addition, we propose the biosynthetic pathway of oxopyrrolidine according to bioinformatic analysis and gene-knockout experiments.

## 2. Results

### 2.1. Isolation and Identification of Marine-Derived Fungi

**Figure 1:**
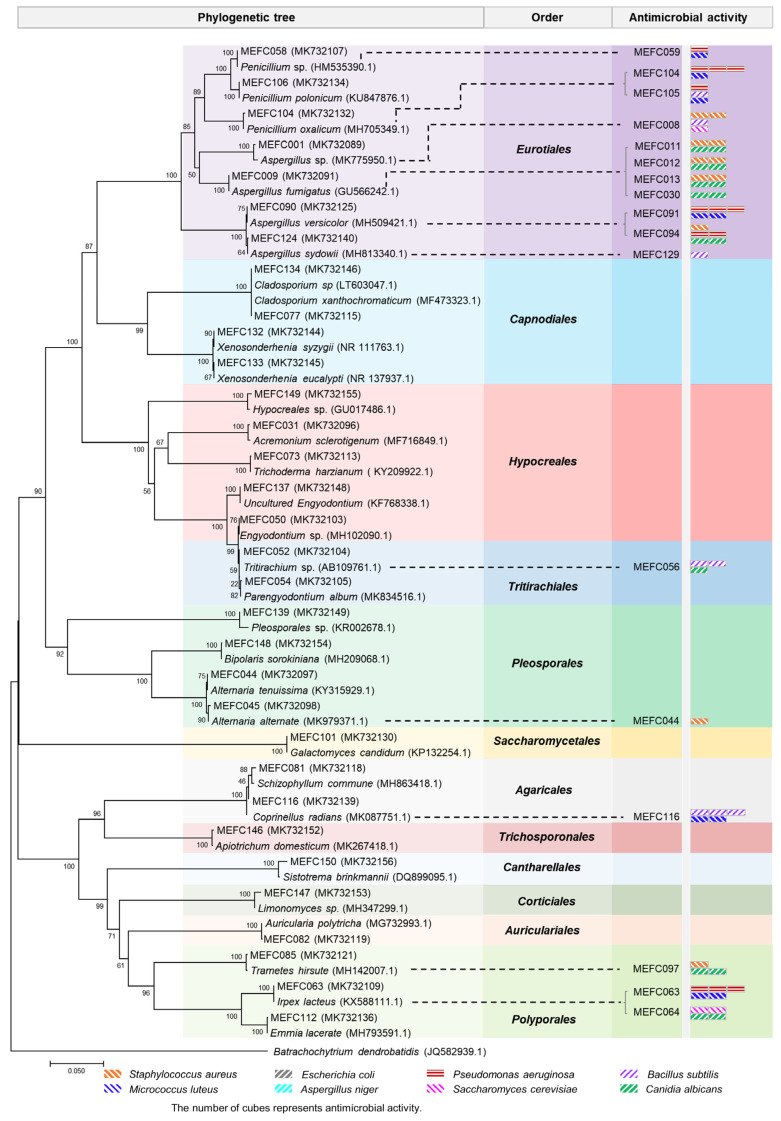
Neighbor-joining phylogenetic tree of the marine-derived fungi in this study.

A total of 151 fungal strains were isolated from marine sediment (collected from Sanggou Bay in Weihai) and seawater (collected from Aoshan Bay in Qingdao). All fungal strains were identified through morphological characteristics and ITS sequence analyses. The neighbor-joining phylogenetic tree revealed that these strains comprised 32 species within 20 genera of 12 taxonomic orders in two phyla (Figure 1 and Table S1). To acquire bioactive metabolites, we cultured all the strains in LPM medium and evaluated the antimicrobial activities of the fermentation extracts against common pathogens, including three Gram-positive strains (*Staphylococcus aureus*, *Micrococcus luteus*, *Bacillus subtilis*) and two Gram-negative strains (*Escherichia coli*, *Pseudomonas aeruginosa*). The fermentation extracts were also tested for antifungal activities against *Aspergillus niger*, *Canidia albicans*, and *Saccharomyces cerevisiae*. The results showed that the extracts of 17 strains (11%) displayed different degrees of antimicrobial activity against the indicator bacteria and fungi (Table S2), among which *P. oxalicum* MEFC104 showed more potent antibacterial activity against *P. aeruginosa* than others. Therefore, *P. oxalicum* MEFC104 was selected for further cultivation and extensive chemical investigation.

### 2.2. Structural Elucidation of Oxopyrrolidine A (**1**) and B (**2**)

**Figure 2:**
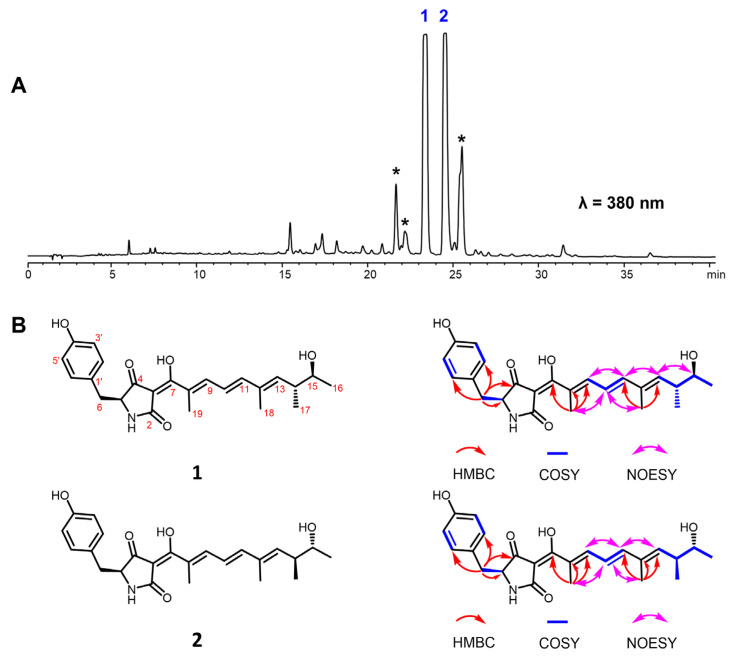
Oxopyrrolidines isolated from *P. oxalicum* MEFC104. (**A**) HPLC profile of *P. oxalicum* MEFC104. The asterisk-marked peaks represent the analogs of **1** and **2**. (**B**) Structures of **1** and **2**.

The HPLC profile showed that the main products in the crude extract of *P. oxalicum* MEFC104 possess similar UV absorption, suggesting that these compounds may be structural analogues (Figure 2A and Figure S1). Compounds **1** and **2**, two new compounds that share the same molecular formula (C_24_H_29_NO_5_), were isolated and characterized. Compound **1** was obtained as a yellow powder, and its molecular formula was determined to be C_24_H_29_NO_5_ with 11 degrees of unsaturation by HRESIMS data. The NMR data of compound **1** (Table 1) indicated a *para*-substituted phenyl moiety, a tetramic acid moiety, and an unsaturated aliphatic side chain, which was similar to those of epicoccarine A [10]. Compared to epicoccarine A, compound **1** showed higher unsaturation and different methylation positions on the aliphatic side chain. Two spin systems of H-9/H-10/H-11 and H-13/H-14/H-15/H-6/H-17 from the ^1^H-^1^H COSY spectrum revealed a long-chain aliphatic alcohol moiety in **1**. The key HMBC correlations of H-18 (*δ*_H_ 1.83, s) to C-11 (*δ*_C_ 146.6) and C-13 (*δ*_C_ 142.7), H-19 (*δ*_H_ 1.89, s) to C-7 (*δ*_C_ 183.7), C-8 (*δ*_C_ 128.2) and C-9 (*δ*_C_ 142.0) suggested that two methyl groups were connected to C-12 and C-8, respectively. The configurations of three *E* double bonds (C-8/C-9, C-10/C-11, C-12/C-13) were determined by NOESY experiments (Figure 2B). Therefore, the planar structure of **1** was established and named oxopyrrolidine A.

Compound **2** was isolated as a yellow powder and deduced as C_24_H_29_NO_5_ based on the HRESIMS spectrum. The ^1^H and ^13^C NMR data of **2** were almost identical to those of **1** (Table 1), and the 2D NMR data revealed a uniform planar structure with **1**, indicating that compound **2** was a diastereomer of **1**. The LC-MS showed that the asterisk-marked compounds have the same molecular weights (411) and similar MS/MS patterns to **1**–**2** (Figure S32). However, we could not acquire enough NMR data to elucidate their structures due to their instability.

**Figure 3:**
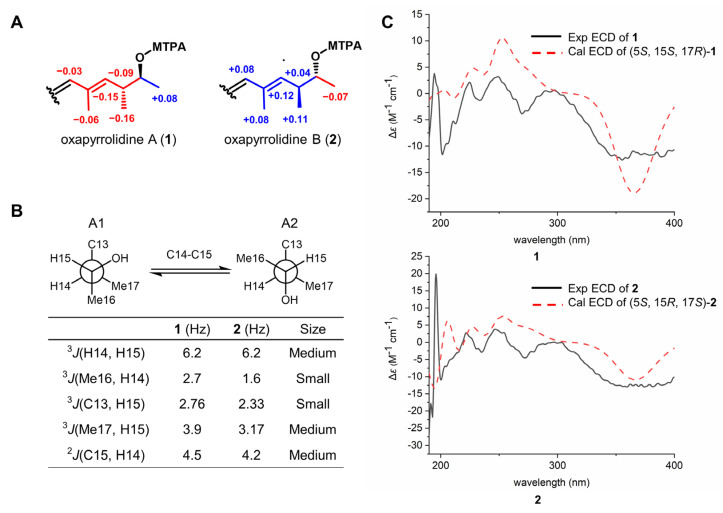
Determination of the absolute configurations of **1** and **2**. (**A**) Δ*δ* (*δ*_S_ − *δ*_R_) values derived from (*S*) and (*R*) MTPA esters of **1** and **2**. (**B**) The relative configuration between H-14/H-15 of **1** and **2** was determined to be a *threo* relationship through ^3^*J*_H14-H15_ and ^2–3^*J*_H-C_ coupling constants. (**C**) The experimental and calculation ECD curves of **1** and **2**.

The absolute configurations of **1** and **2** at C-15 were determined by the modified Mosher’s method. According to the chemical shift difference of MTPA esters of **1** and **2**, the absolute configurations of C-15 were assigned as *S* and *R*, respectively (Figure 3A). Based on this, the configuration of vicinal methine (C-14) was established using *J*-based configuration analysis (Figure S28) [11,12]. The vicinal coupling constants (^3^*J*_C16-H14_, ^3^*J*_C13-H15_, ^3^*J*_C17-H15,_ and ^2^*J*_C15-H14_) were obtained using the HECADE experiment, and a *threo* relationship between H-14 and H-15 was deduced after the analysis of coupling constants (Figure 3B). Thus, the absolute configurations in **1** and **2** at C-14 were assigned as *R* and *S*, respectively. The configuration of the last remaining chiral carbon (C-5) was resolved by ECD calculation. The calculated ECD curves of (5*S*, 15*S*, 17*R*)-**1** and (5*S*, 15*R*, 17*S*)-**2** were similar to their experimental ones (Figure 3C), indicating the 5*S* absolute configuration for both **1** and **2**. This result coincided with the literature that the L-configuration of tyrosine was the precursor in the biosynthesis of *para*-substituted phenyl-tetramic acid derivatives [13].

The antimicrobial activity of compounds **1** and **2** was evaluated. However, compounds **1** and **2** showed no antimicrobial activity against *P. aeruginosa*. This indicated that **1** and **2** were not the active components in the extract of *P. oxalicum* MEFC104. The component with antimicrobial activity may be products at very low levels.

### 2.3. Identification of the Biosynthetic Gene Cluster

**Figure 4:**
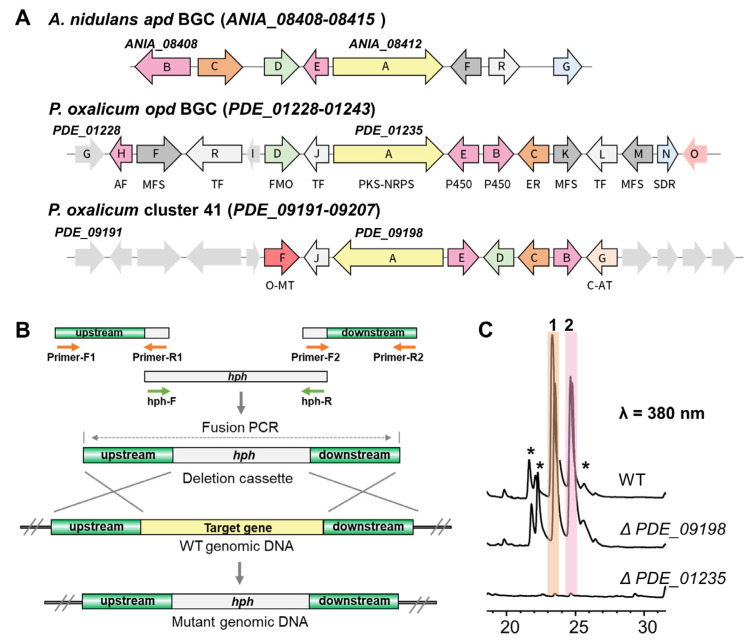
The identification of *opd* BGC. (**A**) Gene schematic of BGCs *apd*, *opd*, and 41. Abbreviations: AF, antifungal proteins; MFS, major facilitator superfamily transporter; TF, transcriptional factor; FMO, flavine-dependent monooxygenase; P450, cytochrome P450; ER, enoyl reductase; SDR, short-chain dehydrogenases/reductases; O-MT, O-methyltransferase; C-AT, chloramphenicol acetyltransferase. (**B**) Schematic illustration of targeted gene disruption by split-marker-based transformation. (**C**) HPLC profiles of extracts from wild type and mutants. The asterisk-marked peaks represent the analogs of **1** and **2**.

To investigate the biosynthetic pathway of oxopyrrolidines, the genome of *P. oxalicum* MEFC104 was sequenced using the Illumina HiSeq X Ten platform. We annotated it based on the genome information of *P. oxalicum* 114-2 (NCBI No. GCA_000346795.1). The prediction accomplished by antiSMASH showed 46 biosynthetic gene clusters (BGCs) containing 60 core enzymes in the genome of *P. oxalicum* MEFC104 (Table S3). Four PKS-NRPS genes, *PDE_01235*, *PDE_04017*, *PDE_04252*, and *PDE_09198*, are located in clusters 7, 20, 22, and 41, respectively. Among them, clusters 7 and 41 show high similarity (33% and 55%) with cluster *apd* in *A. nidulans* (Figure 4A). The *apd* cluster was reported to be responsible for the biosynthesis of aspyridone with a similar structure of 1 [14]. Thus, we deleted *PDE_01235* and *PDE_09198* by gene targeting using *hph* as a selectable marker (Figure 4B). The crude extracts of wild type and mutants were analyzed by HPLC (Figure 4C). Compared to the wild type, mutant Δ*PDE_01235* lost the ability to produce compounds **1**–**2**, while mutant Δ*PDE_09198* did not show any difference. Therefore, cluster 7 containing the PKS-NRPS gene *PDE_01235* was confirmed as responsible for the biosynthesis of oxopyrrolidines and named cluster *opd*.

**Figure 5:**
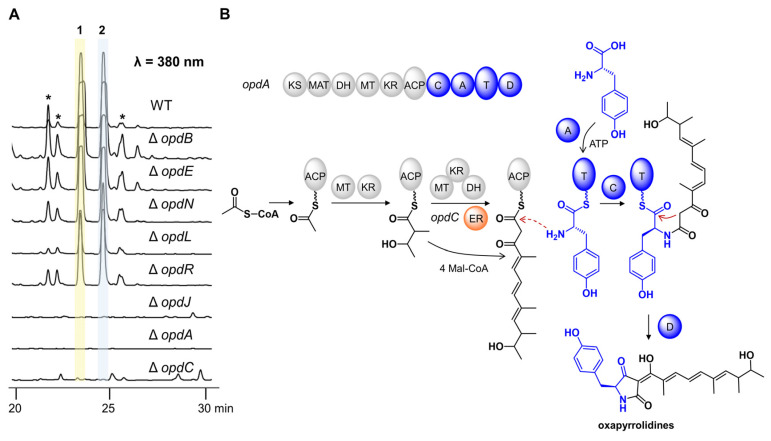
Proposed biosynthetic pathway of oxopyrrolidines by gene-deletion experiments. (**A**) HPLC profiles of *P. oxalicum* WT and deletion mutants. The asterisk-marked peaks represent the analogs of **1** and **2**. (**B**) Proposed biosynthetic pathway of oxopyrrolidines.

Detailed bioinformatic analysis revealed that the *opd* BGC contained 16 genes (Figure 4A, Table S6). To verify their possible functions, all genes were individually inactivated by gene targeting. The crude extracts of all strains were analyzed by HPLC (Figure 5A, Figure S29). OpdJ, OpdL, and OpdR are three transcription factors (TF). Compounds **1**–**2** were abolished in mutant Δ*opdJ*, while no significant differences were observed in mutants Δ*opdL* or Δ*opdR*. The results revealed that OpdJ is the cluster-specific TF responsible for the transcriptional regulation of cluster *opd*. Deleting three MFS transporter encoding genes—*opdF*, *opdK*, and *opdM*—did not affect the biosynthesis of oxopyrrolidines.

The aliphatic side chain of oxopyrrolidines hinted that there were reduction steps in the biosynthetic pathway. However, the OpdA are composed of KS-AT-DH-MT-KR-ACP-C-A-T-D domains, which lack a designated enoyl reductase (ER) domain. This information indicates that there would be a free-standing ER. Eight putative tailoring enzymes were predicted in cluster *opd*, including enoyl reductase OpdC, short-chain dehydrogenase opdN, ligand-binding SRPBCC opdO, two P450s (opdB and opdE), and three hypothetical proteins (opdD, opdG, and opdI). Compared to the wild type, the peaks of compounds **1**–**2** completely disappeared in mutant Δ*opdC*, while no difference was observed in knockout mutants of the other seven genes. No intermediate was accumulated in the culture of mutant Δ*opdC*, indicating that OpdC is a *trans*-acting ER for OpdA and catalyzes the reduction of alkenyl in the assembly process of polyketides. The mechanism was similar to LovC (*trans*-ER) and LovB (PKS) in the biosynthesis of lovastatin [15].

Combining the above data, we propose the biosynthetic pathway of oxopyrrolidines (Figure 5B). The polyketide chain was first assembled by the highly reducing PKS OpdA using acetyl-CoA as the starter unit and five malonyl-CoA as the extender units. The OpdC served as *trans*-acting ER and reduced the terminal alkenyl to alkane. According to the stereochemical rule proposed by Oikawa and coworkers [16], the absolute configurations in C15 and C17 were formed at the chain extension stages in the PKS-NRPS assembly line (Figure S31). The differences between 15*S*, 17*R* in **1** and 15*R*, 17*S* in **2** were generated by non-stereospecific catalysis of the ketoreductase (KR) domain and enoyl reductases. Then the polyketides with specific configurations were transferred to the NRPS module and linked to _L_-tyrosine to form an amide bond. Finally, the oxopyrrolidines were offloaded through a Dieckmann cyclization catalyzed by the terminal D domain to give a tetramic acid moiety.

## 3. Materials and Methods

### 3.1. General Experimental Procedures

The UV spectra were measured on a Beckman DU-800 (Beckman Coulter, Inc., Brea, IN, USA). IR spectra were obtained from a Nicolet iN10 spectrometer (Thermo Fisher Scientific Inc., Waltham, MA, USA) using KBr pellets. NMR spectra were recorded on a Bruker Avance III 600 NMR spectrometer (600 MHz for ^1^H and 150 MHz for ^13^C, Bruker Corporation, Billerica, MA, USA). HRESIMS spectra were acquired from a Dionex Ultimate 3000 system (Thermo Fisher Scientific Inc., MA, USA) coupled with a Bruker Maxis Q-TOF spectrometer (Bruker Corporation, MA, USA) using positive and negative electrospray ionization. CD spectra were recorded on a JASCO J-815-150S circular dichroism spectrometer (Jasco Corp., Tokyo, Japan). The compounds were monitored by an Agilent 1260 system (Agilent Technologies, Santa Clara, CA, USA) equipped with a G1315D photodiode array detector (DAD) using an Agilent ZORBAX Eclipse XDB-C18 column (4.6 × 150 mm, 5 μm) at a flow rate of 1 mL/min. The semi-preparative HPLC experiments were performed on a Waters X Bridge (C18, 5 μm, 150 × 10 mm) column using an Agilent 1260 system at a 2 mL/min flow rate. Silica gel (200–300 mesh, Qing Dao Hai Yang Chemical Group Co., Ltd., Qingdao, China) and octadecyl silyl (ODS) silica gel (40 μm, 120 Å, Ji Nan Bo Na Biological Technology, Jinan, China) were used for column chromatography.

### 3.2. Isolation of Marine-Derived Fungi

The samples, including seawater and marine sediment, were collected from Sanggou Bay and Aoshan Bay, respectively, in the Yellow Sea of China in June 2018. All the samples were diluted with sterile water to three dilutions (1:10, 1:100, and 1:1000), and 150 μL volume of each dilution was plated to triplicate potato dextrose agar (PDA) plates supplemented with streptomycin (25 mg/mL) and ampicillin (25 mg/mL) to inhibit the growth of bacteria. The inoculated plates were placed in an incubator at 30 °C for 1–3 weeks until the morphology of the fungi was observed. The fungi with different morphology and growth characteristics were promptly transferred to new PDA plates during this process. Multiple subcultures or single spores purified all strains to obtain pure colonies. The strains were deposited at the Qingdao Institute of Bioenergy and Bioprocess Technology, Chinese Academy of Sciences, Qingdao, People’s Republic of China.

### 3.3. Screening for Bioactive Marine-Derived Fungal Strains

The marine-derived fungi were inoculated into 50 mL LPM medium (9 g/L glucose, 10 g/L sucrose, 1 g/L yeast extract, 1 g/L peptone, 1 g/L sodium acetate, 0.04 g/L KH_2_PO_4_, 0.1 g/L MgSO_4_, 5 g/L soybean meal, and 1.5 g/L CaCO_3_, pH 6.8) in 250 mL shake flasks for 7 days at 28 °C and 200 rpm. The fermentation broth was extracted three times with twice the volume of ethyl acetate at room temperature and further concentrated in vacuo to obtain crude extracts.

The tested bacteria were cultured in Luria Bertani (LB) broth overnight and were diluted into 10^6^ CFU/mL. Subsequently, 0.1 mL bacterium suspensions were sprayed onto LB plates (Φ 9 cm). The crude extracts were redissolved in DMSO to the concentration of 20 mg/mL and were used as a test solution to evaluate the antibacterial assays. A 5 μL volume of test solution was added to 5 mm sterile filter paper and placed at the LB plates containing bacterial suspensions. Finally, LB plates were incubated at 37 °C for 24 h. DMSO and ampicillin (4 mg/mL) were used as a negative and positive control, respectively. The antibacterial activity of crude extracts was compared by measuring the diameter of the inhibition zone.

The fungi used in antifungal activities were inoculated in PDA at 30 °C to obtain sufficient spores or colonies for preparing a 10^5^ CFU/mL concentration, then 0.1 mL of suspension was sprayed onto the PDA plate. The sterile filter paper with 5 μL crude extracts was added to PDA plates and cultured for 72 h at 30 °C. The DMSO was used as the negative control and amphotericin B (4 mg/mL) as the positive control. The diameter of the inhibition zone was measured to evaluate the antifungal activity. Three repeats were conducted in every antimicrobial experiment.

### 3.4. Culture Conditions

*P. oxalicum* MEFC104 and the mutants used and generated in this study are listed in Table S4. The mycelia used for protoplast transformations were grown in CM medium (3.0 g/L NaNO_3_, 2.6 g/L KCI, 2.6 g/L MgSO_4_·7H_2_O, 7.6 g/L KH_2_PO_4_, 10 g/L glucose, 2 g/L peptones, 1 g/L yeast extract, 1 g/L acid hydrolyzed casein at pH 6.5) [17]. All strains used for fermentation analysis were cultured in LPM medium.

### 3.5. Extraction and Isolation

To purify compounds **1**–**2** for structure characterization, the *P. oxalicum* wild-type strain was fermented in 5 L LPM medium and shaken for 7 days at 28 °C with 220 rpm. The fermentation broth was extracted with ethyl acetate 3 times and concentrated in vacuo to obtain crude extracts. The concentrated organic extracts were subjected to silica gel column chromatography eluting with a gradient mixture of EtOAc/petroleum ether (9:1–1:9, *v*/*v*) to yield three fractions (Fr.1–Fr.3). Fr.2 was further purified by ODS column chromatography using gradient elution with MeOH/H_2_O (1:4–4:1, *v*/*v*) to give four subfractions (Fr.2.1–Fr.2.4). Fr.2.3 was concentrated and purified by HPLC with a semipreparative C18 column eluting with ACN/H_2_O (45:55, *v*/*v*, flow rate, 2.0 mL/min) to afford **1** (8.1 mg, *t*_R_ = 11.6 min) and **2** (4.3 mg, *t*_R_ = 13.6 min).

Compound **1**: Yellow powder; [*α*]^25^_D_ -0.4 (*c*, 0.1, MeOH); UV (MeOH) *λ*_max_ 226 (3.55), 266 (3.71), 392 (5.01); IR (KBr) *ν*_max_ 3413, 2923, 1610, 1542, 1454, 1196, 1132, 634cm^−1^; ^1^H NMR (600 MHz, DMSO-*d*_6_) and ^13^C NMR (150 MHz, DMSO-*d*_6_) spectroscopic data, see Table 1; HRESIMS *m*/*z* 412.2129 [M + H]^+^ (calcd for C_24_H_30_NO_5_ 412.2128).

Compound **2**: Yellow powder; [*α*]^25^_D_ -0.1 (*c*, 0.05, MeOH); UV (MeOH) *λ*_max_ 226 (10.72), 266 (5.89), 392 (6.31); IR (KBr) *ν*_max_ 3413, 2923, 1610, 1542, 1454, 1196, 1132, 634 cm^−1^; ^1^H NMR (600 MHz, DMSO-*d*_6_) and ^13^C NMR (150 MHz, DMSO-d_6_) spectroscopic data, see Table 1; HRESIMS *m*/*z* 412.2125 [M + H]^+^ (calcd for C_24_H_30_NO_5_ 412.2128).

### 3.6. Mosher’s Reactions of Oxopyrrolidine A (**1**) and B (**2**)

To obtain the (*R*)- and (*S*)- MTPA ester derivatives of **1**, 2 mg of **1** was dissolved in 600 μL dry pyridine, then treated with (*S*)-(+)- and (*R*)-(−)-*α*-methoxy-*α*-(trifluoromethyl) phenyl acetyl chloride along with 4-dimethylamino pyridine (5 mg) as a catalyst. The (*S*)- and (*R*)-MTPA ester derivatives of **1** were purified by semipreparative HPLC, and its structure was established by NMR data. The same method was used for the preparation of (*S*)- and (*R*)-MTPA ester derivatives of oxopyrrolidine B (**2**).

(*S*)-MTPA ester of **1**: ^1^H NMR (DMSO-*d*_6_, 600 MHz) *δ*_H_ 5.62 (1H, d, *J* = 10.1 Hz, H-13), 2.82 (1H, m, H-14), 1.80 (3H, s, H-18), 1.29 (3H, d, *J* = 6.4 Hz, H-16), 0.85 (3H, d, *J* = 6.7 Hz, H-17).

(*R*)-MTPA ester of **1**: ^1^H NMR (DMSO-*d*_6_, 600 MHz) *δ*_H_ 5.76 (1H, d, *J* = 9.7 Hz, H-13), 2.91 (1H, m, H-14), 1.85 (3H, s, H-18), 1.21 (3H, d, *J* = 6.3 Hz, H-16), 1.01 (3H, d, *J* = 6.7 Hz, H-17).

(*S*)-MTPA ester of **2**: ^1^H NMR (DMSO-*d*_6_, 600 MHz) *δ*_H_ 5.73 (1H, d, *J* = 9.9 Hz, H-13), 2.90 (1H, m, H-14), 1.84 (3H, s, H-18), 1.21 (3H, d, *J* = 6.3 Hz, H-16), 1.02 (3H, d, *J* = 6.8 Hz, H-17).

(*R*)-MTPA ester of **2**: ^1^H NMR (DMSO-*d*_6_, 600 MHz) *δ*_H_ 5.61 (1H, d, *J* = 9.9 Hz, H-13), 2.87 (1H, m, H-14), 1.76 (3H, s, H-18), 1.29 (3H, d, *J* = 6.4 Hz, H-16), 0.91 (3H, d, *J* = 7.0 Hz, H-17).

### 3.7. Computational Details

Monte Carlo conformational searches were conducted through Spartan’s 10 software using Merck Molecular Force Field (MMFF). The conformers with a Boltzmann population of over 0.6% were chosen for ECD calculations. Then the conformers were optimized at the B3LYP/6-31g (d, p) level in MeOH using the CPCM polarizable conductor calculation model. The theoretical calculation of ECD was conducted in MeOH using time-dependent density functional theory (TD-DFT) at the B3LYP/6-31+g (d, p) level for all conformers of compounds **1** and **2**. Rotatory strengths for a total of 30 excited states were calculated. ECD spectra were generated using the SpecDis 1.6 (University of Würzburg, Würzburg, Germany) and GraphPad Prism 5 (University of California, San Diego, CA, USA) from dipole-length rotational strengths by applying Gaussian band shapes with sigma = 0.3 eV.

### 3.8. Antimicrobial Activity Analysis against P. aeruginosa

*P. aeruginosa* was cultured in LB broth medium overnight and diluted to 10^5^ CFU/mL for antibacterial activity analysis. Subsequently, the two-fold serial dilution of compounds **1** and **2** was redissolved in DMSO to concentrations of 10 mg/mL to 5 μg/mL. Then, a 5 μL test solution was added to 95 μL LB broth containing bacterial suspensions in 96-well plates. Finally, LB broth was incubated at 37 °C for 24 h. DMSO and tetracycline (3–1.5 μg/mL) were used as negative and positive controls, respectively. LB broth was used as a blank. Microplate reader Biotek at 600 nm measured each hole’s absorbance value (OD). The minimum inhibitory concentration (MIC) values of triplicate samples were recorded as the lowest concentration of the compounds with no visible microbial growth.

### 3.9. Protoplast Preparation and Transformation

The protoplast preparation and transformation of *P. oxalicum* were conducted according to a previously reported method with some modifications [18]. The mycelia were collected from CM medium and washed three times with 0.6 M MgSO_4_. The hyphae were transferred into an enzymatic solution (1% snailase, 1% lysing enzymes from *Trichoderma*, and 1% cellulose from *A. niger*) and lysed for 2.5 h. Protoplasts were separated from enzyme solution by filter through 300-mesh nylon and collected in a 50 mL centrifuge tube at 5000 rpm for 20 min. The protoplasts were washed twice with 10 mL of STC buffer (1.2 M Sorbitol, 10 mM Tris-HCl, and 10 mM CaCl_2_) to remove the residual enzyme and then concentrated in 200 μL STC buffer. The gene-targeting cassettes and 20 μL PSTC buffer (40% PEG4000, 1.2 M sorbitol, 10 mM Tris-HCl, and 10 mM CaCl_2_) were added to the protoplasts solution and incubated in the ice bath for 25 min. After incubation, 1 mL of PSTC buffer was added to the protoplast solution and incubated at room temperature for 20 min. The protoplasts were spread on solid PDASH medium (39 g/L PDA, 1.2 M sorbitol, 50 mM hygromycin B). The positive transformants were verified by PCR amplification and purified through single-spore isolation.

### 3.10. Genome Sequencing

The whole genome of MEFC104 was sequenced at Genewiz Ltd. (Suzhou, China) using the Illumina HiSeq X Ten platform. The nucleotide sequence of all genes in *opd* BGC has been deposited into GenBank, and the GenBank accession numbers for *opd*A–*opd*R are ON756038–ON756053.

### 3.11. Construction of P. oxalicum Mutant Strains

To identify the gene cluster involved in oxopyrrolidines biosynthesis, two PKS-NRPS genes (*PDE_01235* and *PDE_09198*) were inactivated by the gene-targeting method. In brief, the 1.2 kb upstream and downstream regions of two PKS-NRPS genes were amplified from the genome DNA and fused with hygromycin resistance gene *hph* by fusion PCR. The gene-targeted cassettes were amplified by PCR using nested primers and then transformed into *P. oxalicum* MEFC104. The positive transformants were confirmed by PCR amplification and cultured in LPM medium for fermentation. To elucidate the biosynthetic pathway of oxopyrrolidines, the genes in cluster *opd* were deleted by the same gene-targeting method.

### 3.12. HPLC and LC-HRMS Analysis of the Metabolites from P. oxalicum Mutant Strains

The fermentation broth of mutants was extracted by EtOAc, and the organic solvent was combined and concentrated in vacuo to obtain crude extract. The extraction was redissolved in MeOH and filtered using 0.22 μm organic membranes to inject into HPLC analysis at a flow rate of 1 mL/min. The solvent gradient system for HPLC was 0–5 min 100–80% A, 5–35 min 80–40% A, 35–50 min 40% A, 50–55 min 40–0% A, 55–60 min 0–100% A, and 60–65 min 100% A (A: 5% ACN/H_2_O with 0.05% formic acid, B: 100% ACN with 0.05% formic acid). The LC-HRMS analysis was carried out on an Agilent C18 column (Eclipse Plus C18 RRHD, 2.1 × 50 mm). The positive mode electrospray ionization was performed with a linear gradient of 10–100% acetonitrile in ddH_2_O with 0.1% formic acid over 6.5 min, followed by 100% acetonitrile for 1.5 min with a flow rate of 0.6 mL/min. The LC-HRMS spectra were recorded on an Agilent 1290 Infinity II coupled to 6545 LC/Q-TOF.

## 4. Conclusions

In summary, we isolated and identified 151 marine-derived filamentous fungi. According to the bioactivity screening and chemical profiles of their crude extracts, *P. oxalicum* MEFC104 was selected for further study, and two new tetramic acid derivatives, oxopyrrolidine A (**1**) and oxopyrrolidine B (**2**) were obtained. The absolute configurations of oxopyrrolidines were determined by modified Mosher’s method, *J*-based configuration analysis, and ECD calculations. Based on bioinformatic analysis and the knockout results of core genes, the *opd* gene cluster was confirmed to be responsible for the biosynthesis of oxopyrrolidines. To verify the function of other genes in the *opd* cluster, all genes were individually deleted in strain MEFC104. The biosynthetic pathway of oxopyrrolidines was proposed. Our study established a link between oxopyrrolidines and the corresponding biosynthesis genes.

## Figures and Tables

**Table 1:** ^1^H (600 MHz) and ^13^C (150 MHz) NMR data for **1** and **2** in DMSO-*d*_6_.

	1			2		
Pos.	*δ*_C_, Type	*δ*_H_ (*J* in Hz)	HMBC	*δ*_C_, Type	*δ*_H_ (*J* in Hz)	HMBC
2	176.9 C			177.1 C		
3	99.1 C			98.5 C		
4	192.0 C			192.3 C		
5	61.3 CH	4.03 (1H, br s)	1′; 6′	61.5 CH	4.02 (1H, s)	
6	36.0 CH_2_	2.85 (2H, m)	5; 1′; 2′; 6′	35.9 CH_2_	2.84 (2H, t, *J* = 4.5 Hz)	4; 5; 1′; 2′; 6′
7	183.7 C			182.7 C		
8	128.2 C			127.5 C		
9	142.0 CH	7.68 (1H, s)		141.7 CH	7.65 (1H, d, *J* = 6.1 Hz)	
10	122.1 CH	6.53 (1H, dd, *J* = 14.9, 11.3 Hz)	8; 9; 11; 12	121.7 CH	6.54 (1H, m)	11
11	146.6 CH	6.64 (1H, m)	9; 10; 12; 13; 18	146.5 CH	6.64 (1H, m)	
12	133.4 C			133.7 C		
13	142.7 CH	5.73 (1H, d, *J* = 9.9 Hz)	11; 14; 15; 17; 18; 19	142.1 CH	5.79 (1H, d, *J* = 9.1 Hz)	11; 14; 15; 17; 18; 19
14	40.8 CH	2.45 (1H, m)	12; 13; 15; 16; 17	39.6 CH	2.54 (1H, m)	12; 13; 15; 16; 17
15	70.0 CH	3.45 (1H, m)	13; 14; 16; 17	69.3 CH	3.57 (1H, m)	13; 14; 16; 17
16	21.6 CH_3_	1.02 (3H, d, *J* = 6.1 Hz)	14; 15	20.6 CH_3_	1.01 (3H, d, *J* = 6.3 Hz)	14; 15
17	16.6 CH_3_	0.97 (3H, d, *J* = 6.6 Hz)	13; 14; 15	16.4 CH_3_	0.96 (3H, d, *J* = 6.8 Hz)	13; 14; 15
18	12.7 CH_3_	1.83 (3H, s)	11; 12; 13; 15; 17	12.4 CH_3_	1.83 (3H, s)	11; 12; 13; 14; 15; 17
19	12.3 CH_3_	1.89 (3H, s)	7; 8; 9; 10; 11	12.2 CH_3_	1.91 (3H, s)	7; 8; 9
1′	125.8 C			125.7 C		
2′	130.8 CH	6.93 (1H, d, *J* = 7.9 Hz)	6; 3′; 4′; 5′; 6′	130.6 CH	6.94 (2H, d, *J* = 8.0 Hz)	6; 3′; 4′; 5′; 6′
3′	114.8 CH	6.61 (1H, d, *J* = 7.9 Hz)	1′; 4′; 5′	114.6 CH	6.62 (1H, d, *J* = 8.0 Hz)	1′; 4′; 5′
4′	156.0 C			155.8 C		
5′	114.8 CH	6.61 (1H, d, *J* = 7.9 Hz)	1′; 3′; 4′	114.6 CH	6.62 (1H, d, *J* = 8.0 Hz)	1′; 3′; 4′
6′	130.8 CH	6.93 (1H, d, *J* = 7.9 Hz)	6; 2′; 3′; 4′; 5′	130.6 CH	6.94 (2H, d, *J* = 8.0 Hz)	6; 2′; 3′; 4′; 5′
	15-OH	3.51 (1H, s)	15			

## Data Availability

Data are contained within the article and Supplementary Material.

## Supplementary Materials

The following are available online at https://www.mdpi.com/article/10.3390/md20080523/s1, Table S1: Phylogenetic affiliations of the representative isolates of 32 species, Table S2: Antimicrobial activities of the crude extracts from the marine-derived fungi, Table S3: BGCs in the genome of *P. oxalicum* MEFC104, Table S4: The strains used in gene-deletion experiments, Table S5: The oligonucleotides used in this study, Table S6: Functional annotation of genes in the *opd* gene cluster, Figure S1: The HPLC profile of crude extracts in *P. oxalicum* MEFC104, Figures S2–S27: HRESIMS, UV, ECD and NMR spectra of compounds **1** and **2**, Figure S28. *J*-based analysis includes all conformer pairs that produce a medium ^3^*J*(H_14_-H_15_), Figure S29. HPLC profiles of extracts from wild-type and mutants, Figure S30. Extracted ion chromatograms of extracts from wild-type and mutants, Figure S31. Stereochemical course of fungal reduced polyketides, Figure S32. MS/MS data of oxopyrrolidines (**1**–**2**) and their analogues. Figure S33. Fragmentation pattern of oxopyrrolidines based on MS/MS data.
