# Supplementary material for: Identification of PKS-NRPS Hybrid Metabolites in Marine-Derived Penicillium oxalicum
Source: Mar Drugs. 2022 Aug 16;20(8):523. doi: 10.3390/md20080523 (PMC9409647; doi:10.3390/md20080523)
Supplement: Supplementary file 1 [file marinedrugs-20-00523-s001.zip › marinedrugs-1784987-supplementary.pdf]

Supporting Information

Identification of PKS-NRPS Hybrid Metabolites in Marine-Derived *Penicillium oxalicum*

Hongcheng Li [1,2,3†], Wei Zhang [1,4,5,6†], Xuan Zhang[1,4,5], Shen Tang [1,3], Ping Men [1,4,5,6], Mengyi Xiong[1,4,5], Zhimin Li [3], Yongyu Zhang [1], Xuenian Huang [1,4,5,6*] and Xuefeng Lu [1,4,5,6,7,8*]

[1] Shandong Provincial Key Laboratory of Synthetic Biology, Qingdao Institute of Bioenergy and Bioprocess Technology, Chinese Academy of Sciences, Qingdao 266101, China

[2] School of Medicine and Pharmacy, Ocean University of China, Qingdao 266003, China

[3] Jiangxi Engineering Laboratory for the Development and Utilization of Agricultural Microbial Resources, College of Bioscience and Bioengineering, Jiangxi Agricultural University, Nanchang 330045, China

[4] Shandong Energy Institute, Qingdao 266101, China

[5] Qingdao New Energy Shandong Laboratory, Qingdao 266101, China

[6] University of Chinese Academy of Sciences, Beijing 100049, China

[7] Marine Biology and Biotechnology Laboratory, Qingdao National Laboratory for Marine Science and Technology, Qingdao, 266101, China

[8] Key Laboratory of Biofuels, Qingdao Institute of Bioenergy and Bioprocess Technology, Chinese Academy of Sciences, Qingdao 266101, China

* Correspondence: huangxn@qibebt.ac.cn; lvxf@qibebt.ac.cn

† H.L. and W.Z. contributed equally to this work.

Table of contents

Table of contents	Page
Table S1. Phylogenetic affiliations of the representative isolates of 32 species	S4
Table S2. Antimicrobial activities of the crude extracts from the marine-derived fungi	S5
Table S3. BGCs in the genome of *P. oxalicum* MEFC104	S6
Table S4. The strains used in gene deletion experiments	S7
Table S5. The oligonucleotides used in this study	S8
Table S6. Functional annotation of genes in *opd* gene cluster	S11
Figure S1. The HPLC profile of crude extracts in *P. oxalicum* MEFC104	S12
Figure S2. HRESIMS spectrum of oxapyrrolidine A (1)	S13
Figure S3. ^{1}H NMR spectrum of oxapyrrolidine A (1) in DMSO-d_6	S14
Figure S4. ^{13}C NMR spectrum of oxapyrrolidine A (1) in DMSO-d_6	S15
Figure S5. DEPT 135 spectrum of oxapyrrolidine A (1) in DMSO-d_6	S16
Figure S6. HSQC spectrum of oxapyrrolidine A (1) in DMSO-d_6	S17
Figure S7. ^{1}H-^{1}H COSY spectrum of oxapyrrolidine A (1) in DMSO-d_6	S18
Figure S8. HMBC spectrum of oxapyrrolidine A (1) in DMSO-d_6	S19
Figure S9. NOESY spectrum of oxapyrrolidine A (1) in DMSO-d_6	S20
Figure S10. Key regions of ^{1}H spectrum of Mosher's ester of oxapyrrolidine A (1) in DMSO-d_6	S21
Figure S11. HECADE spectrum of oxapyrrolidine A (1) in DMSO-d_6	S22
Figure S12. ECD spectrum of oxapyrrolidine A (1)	S23
Figure S13. UV spectrum of oxapyrrolidine A (1)	S24
Figure S14. IR spectrum of oxapyrrolidine A (1)	S25
Figure S15. HRESIMS spectrum of oxapyrrolidine B (2)	S26
Figure S16. ^{1}H NMR spectrum of oxapyrrolidine B (2) in DMSO-d_6	S27
Figure S17. ^{13}C NMR spectrum of oxapyrrolidine B (2) in DMSO-d_6	S28
Figure S18. DEPT 135 spectrum of oxapyrrolidine B (2) in DMSO-d_6	S29
Figure S19. HSQC spectrum of oxapyrrolidine B (2) in DMSO-d_6	S30
Figure S20. ^{1}H-^{1}H COSY spectrum of oxapyrrolidine B (2) in DMSO-d_6	S31

Figure S21. HMBC spectrum of oxapyrrolidine B (2) in DMSO-d_6 S32

Figure S22. NOESY spectrum of oxapyrrolidine B (2) in DMSO-d_6 S33

Figure S23. Key regions of ^{1}H spectrum of Mosher's ester of oxapyrrolidine B (2) in DMSO-d_6 S34

Figure S24. HECADE spectrum of oxapyrrolidine B (2) in DMSO-d_6 S35

Figure S25. ECD spectrum of oxapyrrolidine B (2) S36

Figure S26. UV spectrum of oxapyrrolidine B (2) S37

Figure S27. IR spectrum of oxapyrrolidine B (2) S38

Figure S28. J-based analysis including all conformer pairs that produce a medium $^3J_{(H14-H15)}$ S39

Figure S29. HPLC profiles of extracts from wild type and mutants S40

Figure S30. Extracted ion chromatograms of extracts from wild type and mutants S41

Figure S31. Stereochemical course of fungal reduced polyketides S42

Figure S32. MS/MS data of oxapyrrolidines (1-2) and asterisk-marked compounds. S43

Figure S33. Fragmentation pattern of oxapyrrolidines based on MS/MS data. S44

Table S1. Phylogenetic affiliations of the representative isolates of 32 species

Isolate ID	Order	Genus	Accession number	Closest identified relative	Identity (%)	Overlap (bp)
MEFC001	*Eurotiales*	*Aspergillus*	MK732089	*Aspergillus* sp. (MK775950.1)	99	1112
MEFC009			MK732091	*Aspergillus fumigatus* (GU566242.1)	99	1153
MEFC090			MK732125	*Aspergillus versicolor* (MH509421.1)	100	1014
MEFC124			MK732140	*Aspergillus sydowii* (MH813340.1)	100	1026
MEFC058		*Penicillium*	MK732107	*Penicillium* sp. (HM535390.1)	100	1048
MEFC104			MK732132	*Penicillium oxalicum* (MH705349.1)	100	1044
MEFC106			MK732134	*Penicillium polonicum* (KU847876.1)	100	1051
MEFC031	*Hypocreales*	*Acremonium*	MK732096	*Acremonium sclerotigenum* (MF716849.1)	100	1037
MEFC050		*Engyodontium*	MK732103	*Engyodontium* sp. (MH102090.1)	100	1123
MEFC137			MK732148	*Uncultured Engyodontium* (KF768338.1)	100	1127
MEFC073		*Trichoderma*	MK732113	*Trichoderma harzianum* (KY209922.1)	100	1131
MEFC149			MK732155	*Hypocreales* sp. (GU017486.1)	99	1020
MEFC044	*Pleosporales*	*Alternaria*	MK732097	*Alternaria tenuissima* (KY315929.1)	100	1050
MEFC045			MK732098	*Alternaria alternate* (MK979371.1)	99	1020
MEFC139			MK732149	*Pleosporales* sp. (KR002678.1)	100	968
MEFC148		*Cochliobolus*	MK732154	*Bipolaris sorokiniana* (MH209068.1)	100	1059
MEFC077	*Capnodiales*	*Cladosporium*	MK732115	*Cladosporium xanthochromaticum* (MF473323.1)	100	1061
MEFC134			MK732146	*Cladosporium* sp. (LT603047.1)	100	996
MEFC132		*Xenosonderhenia*	MK732144	*Xenosonderhenia syzygii* (NR_111763.1)	94	785
MEFC133			MK732145	*Xenosonderhenia eucalypti* (NR_137937.1)	94	782
MEFC052	*Tritirachiales*	*Tritirachium*	MK732104	*Tritirachium* sp. (AB109761.1)	100	1123
MEFC054			MK732105	*Parengyodontium album* (MK834516.1)	99	1077
MEFC101	*Saccharomycetales*	*Geotrichum*	MK732130	*Galactomyces candidum* (KP132254.1)	97	623
MEFC063	*Polyporales*	*Irpex*	MK732109	*Irpex lacteus* (KX588111.1)	100	1208
MEFC085		*Trametes*	MK732121	*Trametes hirsute* (MH142007.1)	100	1118
MEFC112		*Emmia*	MK732136	*Emmia lacerate* (MH793591.1)	100	1158
MEFC081	*Agaricales*	*Schizophyllum*	MK732118	*Schizophyllum commune* (MH863418.1)	100	1147
MEFC116		*Coprinellus*	MK732139	*Coprinellus radians* (MK087751.1)	100	1225
MEFC082	*Auriculariales*	*Auricularia*	MK732119	*Auricularia polytricha* (MG732993.1)	100	1133
MEFC147	*Corticiales*	*Limonomyces*	MK732153	*Limonomyces* sp. (MH347299.1)	100	1162
MEFC150	*Cantharellales*	*Sistotrema*	MK732156	*Sistotrema brinkmannii* (DQ899095.1)	99	1273
MEFC146	*Trichosporonales*	*Apiotrichum*	MK732152	*Apiotrichum domesticum* (MK267418.1)	100	965

Table S2. Antimicrobial activities of the crude extracts from the marine-derived fungi

Number	Species	*S. aureus*	*E. coli*	*P. aeruginosa*	*B. subtilis*	*M. luteus*	*A. niger*	*S. cerevisiae*	*C. albicans*
MEFC008	*Aspergillus terreus*	++	-	-	+	-	-	+	-
MEFC011	*Aspergillus fumigatus*	++	-	-	-	-	-	-	++
MEFC012	*Aspergillus fumigatus*	++	-	-	-	-	-	-	++
MEFC013	*Aspergillus fumigatus*	++	-	-	-	-	-	-	++
MEFC030	*Aspergillus fumigatus*	-	-	-	-	-	-	-	++
MEFC044	*Alternaria alternata*	+	-	-	-	-	-	-	-
MEFC056	*Tritirachium*	-	-	-	+	-	-	-	++
MEFC059	*Penicillium chrysogenum*	-	-	+	-	+	-	-	-
MEFC063	*Irpex lacteus*	-	-	+++	-	++	-	-	-
MEFC064	*Irpex lacteus*	-	-	-	-	-	-	++	++
MEFC091	*Aspergillus versicolor*	-	-	+++	-	++	-	-	-
MEFC094	*Aspergillus versicolor*	+	-	++	-	-	-	-	++
MEFC097	*Trametes hirsuta*	+	-	-	-	-	-	-	++
MEFC104	*Penicillium oxalicum*	-	-	+++	-	+	-	-	-
MEFC105	*Penicillium oxalicum*	-	-	+	++	+	-	-	-
MEFC116	*Coprinellus radians*	-	-	-	+++	++	-	-	-
MEFC129	*Aspergillus sydowii*	-	-	-	+	-	-	-	-

Positive control: Ampicillin (bacterial, 4.0 mg/mL); Amphotericin B (fungi, 4.0 mg/mL); negative control: dimethyl sulfoxide (DMSO); – no antimicrobial activity; + weak inhibitory activity (inhibition zone between 5 and 10 mm); ++ moderate inhibitory activity (inhibition zone between 10 and 15 mm); +++ strong inhibitory activity (inhibition zone > 15 mm).

Table S3. BGCs in the genome of *P. oxalicum* MEFC104

No.	Locus tag	Type	Products	No.	Locus tag	Type	Products
1	PDE_00149	Terpene		21	PDE_04074	NRPS	
2	PDE_00404	Betalactone		22	PDE_04252	PKS-NRPS	
	PDE_00405	Betalactone		23	PDE_04436	NRPS-like	
3	PDE_00789	NRPS			PDE_04438	NRPS-like	
	PDE_00793	NRPS			PDE_04441	T1 PKS	
	PDE_00797	Indole		24	PDE_04543	NRPS	
	PDE_00807	Indole		25	PDE_05564	NRPS-like	
	PDE_00810	NRPS		26	PDE_05889	Indole	
4	PDE_01066	Indole	Roquefortine C	27	PDE_06013	Terpene	
	PDE_01071	NRPS		28	PDE_06094	NRPS-like	
	PDE_01075	Terpene		29	PDE_06154	Terpene	
	PDE_01077	NRPS		30	PDE_06206	NRPS	
5	PDE_01185	NRPS-like		31	PDE_06630	NRPS-like	
6	PDE_01212	NRPS		32	PDE_07005	NRPS	
7	PDE_01235	PKS-NRPS		33	PDE_07163	NRPS	
8	PDE_01430	Terpene		34	PDE_07205	Terpene	
	PDE_01432	NRPS		35	PDE_07373	NRPS-like	
9	PDE_01655	Terpene		36	PDE_08027	Terpene	
10	PDE_01717	Terpene		37	PDE_08071	Terpene	
11	PDE_02026	Siderophore		38	PDE_08155	NRPS-like	
12	PDE_02110	T3 PKS		39	PDE_08329	NRPS-like	
13	PDE_02131	NRPS		40	PDE_08437	NRPS-like	
	PDE_02141	T1 PKS		41	PDE_09198	PKS-NRPS	
14	PDE_02203	NRPS-like		42	PDE_09237	T1 PKS	
15	PDE_02610	NRPS		43	PDE_09491	T1 PKS	Naphthopyrone
16	PDE_03304	NRPS-like		44	PDE_09561	Terpene	Clavaric acid
17	PDE_03416	NRPS		45	PDE_09740	Indole	Emericellin
18	PDE_03455	T1 PKS			PDE_09743	NRPS-like	
19	PDE_03926	T1 PKS		46	PDE_10001	Terpene	
20	PDE_04017	PKS-NRPS	Oxaleimide C		PDE_10006	T1 PKS	

Table S4. The strains used in gene deletion experiments

Strain	Genotype	Reference
MEFC104-WT	*P. oxalicum* MEFC104	This study
MEFC104-Δ*apnA*	*P. oxalicum* MEFC104, *ΔapnA::hph*$^{-}$	This study
MEFC104-Δ*opdA*	*P. oxalicum* MEFC104, *ΔopdA::hph*$^{-}$	This study
MEFC104-Δ*opdB*	*P. oxalicum* MEFC104, *ΔopdB::hph*	This study
MEFC104-Δ*opdC*	*P. oxalicum* MEFC104, *ΔopdC::hph*	This study
MEFC104-Δ*opdD*	*P. oxalicum* MEFC104, *ΔopdD::hph*	This study
MEFC104-Δ*opdE*	*P. oxalicum* MEFC104, *ΔopdE::hph*	This study
MEFC104-Δ*opdF*	*P. oxalicum* MEFC104, *ΔopdF::hph*	This study
MEFC104-Δ*opdG*	*P. oxalicum* MEFC104, *ΔopdG::hph*	This study
MEFC104-Δ*opdH*	*P. oxalicum* MEFC104, *ΔopdH::hph*	This study
MEFC104-Δ*opdI*	*P. oxalicum* MEFC104, *ΔopdI::hph*	This study
MEFC104-Δ*opdJ*	*P. oxalicum* MEFC104, *ΔopdJ::hph*	This study
MEFC104-Δ*opdK*	*P. oxalicum* MEFC104, *ΔopdK::hph*	This study
MEFC104-Δ*opdL*	*P. oxalicum* MEFC104, *ΔopdL::hph*	This study
MEFC104-Δ*opdM*	*P. oxalicum* MEFC104, *ΔopdM::hph*	This study
MEFC104-Δ*opdN*	*P. oxalicum* MEFC104, *ΔopdN::hph*	This study
MEFC104-Δ*opdO*	*P. oxalicum* MEFC104, *ΔopdO::hph*	This study
MEFC104-Δ*opdR*	*P. oxalicum* MEFC104, *ΔopdR::hph*	This study

Table S5. The oligonucleotides used in this study

Primers	Sequence (5′-3′)
hph-F	TTCGGGATCGCAAGCGTAAAG
hph-R	CAATTATCTTTGCGAACCCAGG
U-01235-F1	AGGAGGCCACCTTTACCAAT
U-01235-R1	CTTTACGCTTGCGATCCCGAATCTCTGTGACTGATTCCCAC
D-01235-F1	CCTGGGTTCGCAAAGATAATTGGCTCTACAGGCCATCATTCT
D-01235-R1	TTGAACAGTGGGAGGTCACC
U-01235-F	ATCCAATCAAGGATCGCTGC
D-01235-R	GGGGCTGAACAACAAGAGTC
U-09198-F1	CTCGCCATCAAGAATCGTGT
U-09198-R1	CTTTACGCTTGCGATCCCGAATAAGCAGTATCGGTCCTCAGG
D-09198-F1	CCTGGGTTCGCAAAGATAATTGCGGGCTGTCGATTCTTATTTG
D-09198-R1	GCAACAAACGGTTCCAAGCC
U-09198-F	GTACGTCGGATCCGTGAAGAC
D-09198-R	CAGTTGTTTCACATCTGTCGC
U-01228-F1	ATGCTGAGCGCAATGATCTC
U-01228-R1	CTTTACGCTTGCGATCCCGAATTTAGACTTGGTTGGTTTCT
D-01228-F1	CCTGGGTTCGCAAAGATAATTGGGATAAATTCGTTCTC
D-01228-R1	GAGAATCGCGAAGTCTCTGG
U-01228-F	AAGCGGCATGTAGCCCTGAT
D-01228-R	TATGCGACGGAAATCACCGG
U-01229-F1	TCCATTATGCCGGGAGTTCA
U-01229-R1	CTTTACGCTTGCGATCCCGAAATTGAGAGAGTTGAACGTGA
D-01229-F1	CCTGGGTTCGCAAAGATAATTGGCTTCTATGAGCGTCAAGCC
D-01229-R1	CAAAGGTGCGATGAGCAAGC
U-01229-F	TTGAAAGGCTCATATGGCTC
D-01229-R	AACTTCATCACCTTGCAGAC
U-01230-F1	ATAGCTGGAATGGCCCACC
U-01230-R1	CTTTACGCTTGCGATCCCGAACCCTTCAACTGGTTCGTTT
D-01230-F1	CCTGGGTTCGCAAAGATAATTGATCGGCCTTGGGCCCGTCC
D-01230-R1	CCTTGTTGGTCTCATATCCA
U-01230-F	CAGCGGGTCCGATTCATACCT
D-01230-R	CTATATGTCTTCACCTGTCG
U-01231-F1	CCTCGTTGCCTTTCTCTAAA
U-01231-R1	CTTTACGCTTGCGATCCCGAATGAAACTGGAATTTGACGT
D-01231-F1	CCTGGGTTCGCAAAGATAATTGACCCTCGATTCTACCTGT
D-01231-R1	GTTTACATTGCTGCCTGCAA

U-01231-F	CAATTGACCCAGCCAAATGAG
D-01231-R	TTGTTGGACAGGAGTTGTGC
U-01232-F1	ACGCAGCTTGAAGAAGAGGCTT
U-01232-R1	CTTTACGCTTGCGATCCCGAATGTATCGGAGCCCATATTAT
D-01232-F1	CCTGGGTTCGCAAAGATAATTGAGCTTCTCAGTCAGGAGA
D-01232-R1	CGATCACCATCGATGAGATC
U-01232-F	TCAGACAACTTGGATGACCTC
D-01232-R	ATACGTGGACAGTGCATGAC
U-01233-F1	TGACAGCTGATTGCATCACG
U-01233-R1	CTTTACGCTTGCGATCCCGAACCCTGTGTGGGGATTTCGG
D-01233-F1	CCTGGGTTCGCAAAGATAATTGGACCGTCATCTGGTGGGTC
D-01233-R1	GCAGGCAGCAATGTAAACTG
U-01233-F	CGGTTTCGACTGTGTCAAAG
D-01233-R	AAACGTGACAGGAGAGATGA
U-01234-F1	TGCAACGGTTCGAACAGACCA
U-01234-R1	CTTTACGCTTGCGATCCCGAATTCGGGTGCTAGAGCTGCC
D-01234-F1	CCTGGGTTCGCAAAGATAATTGGGCATTAAATAGCGTCCTG
D-01234-R1	GTGGTGATTGGTGACCAAGCA
U-01234-F	ATGGGCGGAGCGTCAAAGA
D-01234-R	GTGCGGAAGGTTATACCGCA
U-01236-F1	TTTGACATCGGTCGAGCCAT
U-01236-R1	CTTTACGCTTGCGATCCCGAAAATCACACGTGCTCCCTGG
D-01236-F1	CCTGGGTTCGCAAAGATAATTGGATATAGTCCAGGTTACC
D-01236-R1	AAGCTTCCGAGCCAGAGTCT
U-01236-F	CCAGAAGGATACGTTGTTGG
D-01236-R	AATGCACCGCGGATGCGAA
U-01237-F1	ACCATTATCGCGCCGGTCT
U-01237-R1	CTTTACGCTTGCGATCCCGAAGAGTCACCAGGAATCTCTG
D-01237-F1	CCTGGGTTCGCAAAGATAATTGAACCTAGCAAGCAATCCC
D-01237-R1	TGGGTGTGGAGAGAGACCAT
U-01237-F	TGGCCAGACCACAAGGTAGT
D-01237-R	CCGCGATGAGAATCCGTTCA
U-01238-F1	AAATCATGGACCTGGTCAGG
U-01238-R1	CTTTACGCTTGCGATCCCGAAAACTCGGGTAACGGGTGCCCT
D-01238-F1	CCTGGGTTCGCAAAGATAATTGTGGTGGGTTGTCCAGTGT
D-01238-R1	TGTTCAGCGGAGTAGCGTAT
U-01238-F	ATCGTTCGCCAATGGTCACC
D-01238-R	ACGAGGTCGGATATCGGATG

U-01239-F1	ACTCCTAAGCCATTTCCCGT
U-01239-R1	CTTTACGCTTGCGATCCCGAAACAGAGCACGGAGGATATC
D-01239-F1	CCTGGGTTCGCAAAGATAATTGTTCACCCTCCTGTTTGGC
D-01239-R1	CCGCATTCGACGATGCCTAT
U-01239-F	AGGTCAGCAGCGACATCAAG
D-01239-R	ATTCCTTGATCCTCAACGG
U-01240-F1	TCAAGGCTGGACTCGCTATG
U-01240-R1	CTTTACGCTTGCGATCCCGAAAAAGCGCGCAGATATTCTTC
D-01240-F1	CCTGGGTTCGCAAAGATAATTGCTCCCAGCGGCTTGATCAT
D-01240-R1	TGGGATCGGATTCCAGAAGA
U-01240-F	TGGCACCTTGCTTCTTAGGG
D-01240-R	CCAACAGCATCAAAAACACG
U-01241-F1	AATGGGTGATCTGGATTGC
U-01241-R1	CTTTACGCTTGCGATCCCGAAGAAGAAAGAGAGGAAGGTC
D-01241-F1	CCTGGGTTCGCAAAGATAATTGGCTACGAATCTTTGGTAGG
D-01241-R1	CGCGATAGAGGGATTCTCAA
U-01241-F	ATGGGGAATGATGATGCAGAAGC
D-01241-R	GGAGTTGAGATACAGGAAGTACC
U-01242-F1	GGTAAGTGGAAATTGTGGG
U-01242-R1	CTTTACGCTTGCGATCCCGAAGGCGTAATGAAATTTGGCG
D-01242-F1	CCTGGGTTCGCAAAGATAATTGAGGGCGATATTCTCCCGT
D-01242-R1	AGTACGACCGTAAGAAGGTG
U-01242-F	TCCATATCTGTCTCAACGCC
D-01242-R	TGTGGTACTTCCTGTATCTC
U-01243-F1	ACTCCCGCAGAAGTGCTGTC
U-01243-R1	CTTTACGCTTGCGATCCCGAATGGAATGGATGAGCTTTGC
D-01243-F1	CCTGGGTTCGCAAAGATAATTGGCTCAAGAAGCTGAGGTG
D-01243-R1	GGTAGGAGCAGCCTCCTATATA
U-01243-F	GTAGTTTCTGCCGTATGCTG
D-01243-R	TCCTCTTAGTATCGATCGGC

Table S6. Functional annotation of genes in *opd* gene cluster

Gene tag	Locus tag	Putative function	Characterized homolog	Protein identity
opdG	PDE_01228	Hypothetical protein		
opdH	PDE_01229	Antifungal protein		
opdF	PDE_01230	MFS transporter	*apdF*(ANIA_08413)	32.86%
opdR	PDE_01231	Transcription factor	*apdR*(ANIA_08414)	30.79%
opdI	PDE_01232	-	-	
opdD	PDE_01233	Hypothetical protein	*apdD*(ANIA_08410)	51.08
opdJ	PDE_01234	Transcription factor	-	
opdA	PDE_01235	PKS-NRPS	*apdA*(ANIA_08412)	45.60%
opdE	PDE_01236	Cytochrome P450	*apdE*(ANIA_08411)	54.98%
opdB	PDE_01237	Cytochrome P450	*apdB*(ANIA_08408)	37.36%
opdC	PDE_01238	Enoyl reductase	*apdC*(ANIA_08409)	49.27%
opdK	PDE_01239	MFS transporter	*apdF*(ANIA_08413)	36.19%
opdL	PDE_01240	Transcription factor	-	
opdM	PDE_01241	MFS transporter	-	
opdN	PDE_01242	Short-chain dehydrogenase	-	
opdO	PDE_01243	Ligand-binding SRPBCC	-	

Figure S1. (A) The HPLC profile of crude extracts in *P. oxalicum* MEFC104. (B) The UV absorbs of compounds at 15-20 min.

A

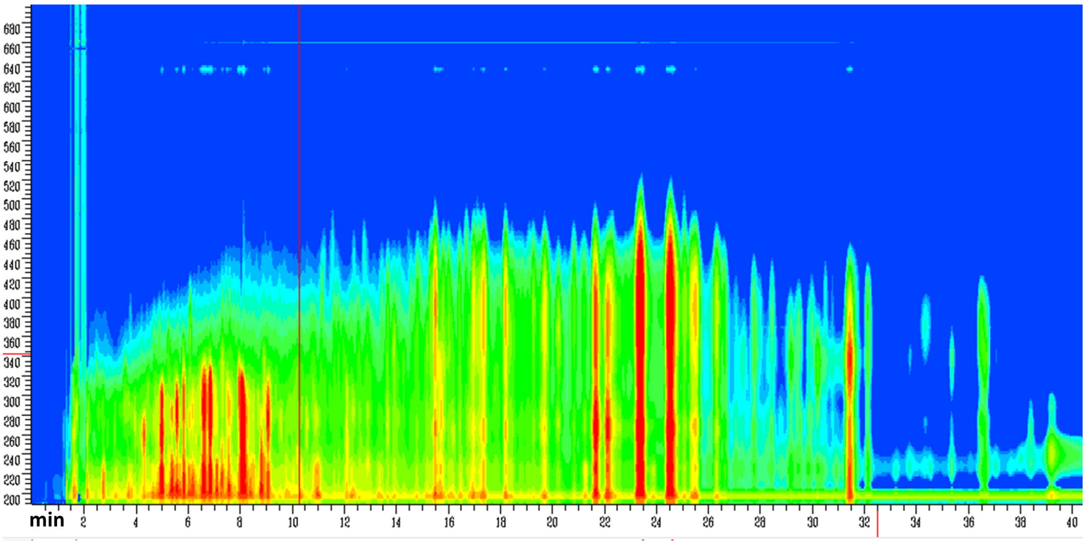

B

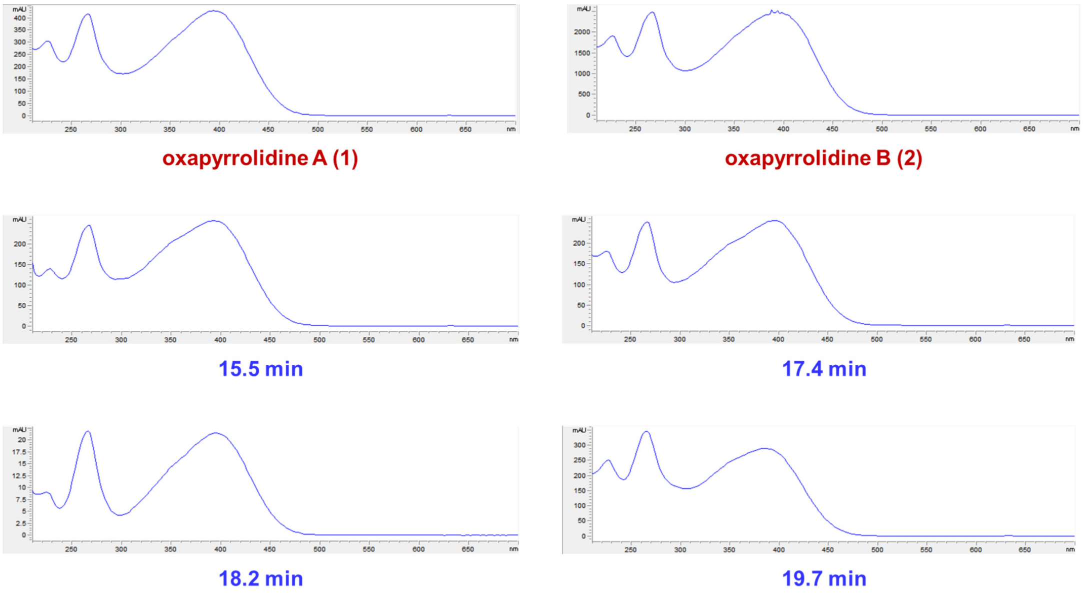

Figure S2. HRESIMS spectrum of oxapyrrolidine A (**1**)

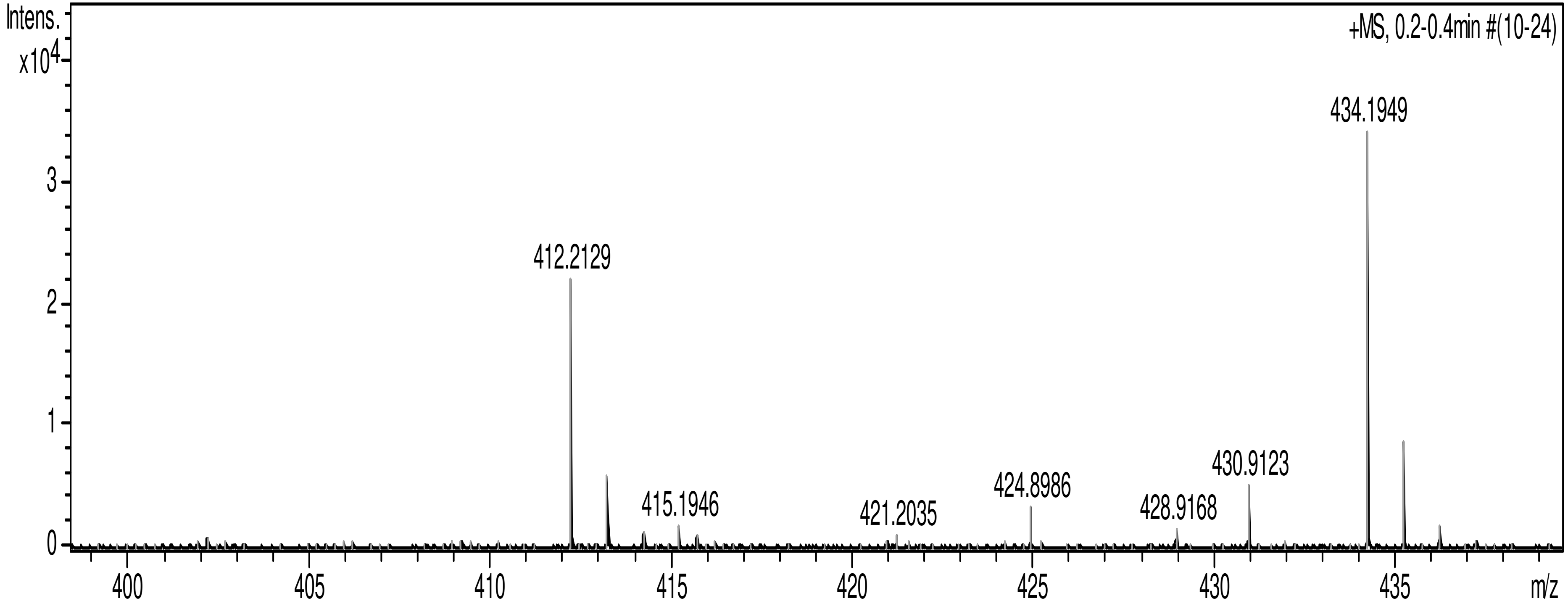

Figure S3. ^{1}H NMR spectrum of oxapyrrolidine A (**1**) in DMSO-d_6

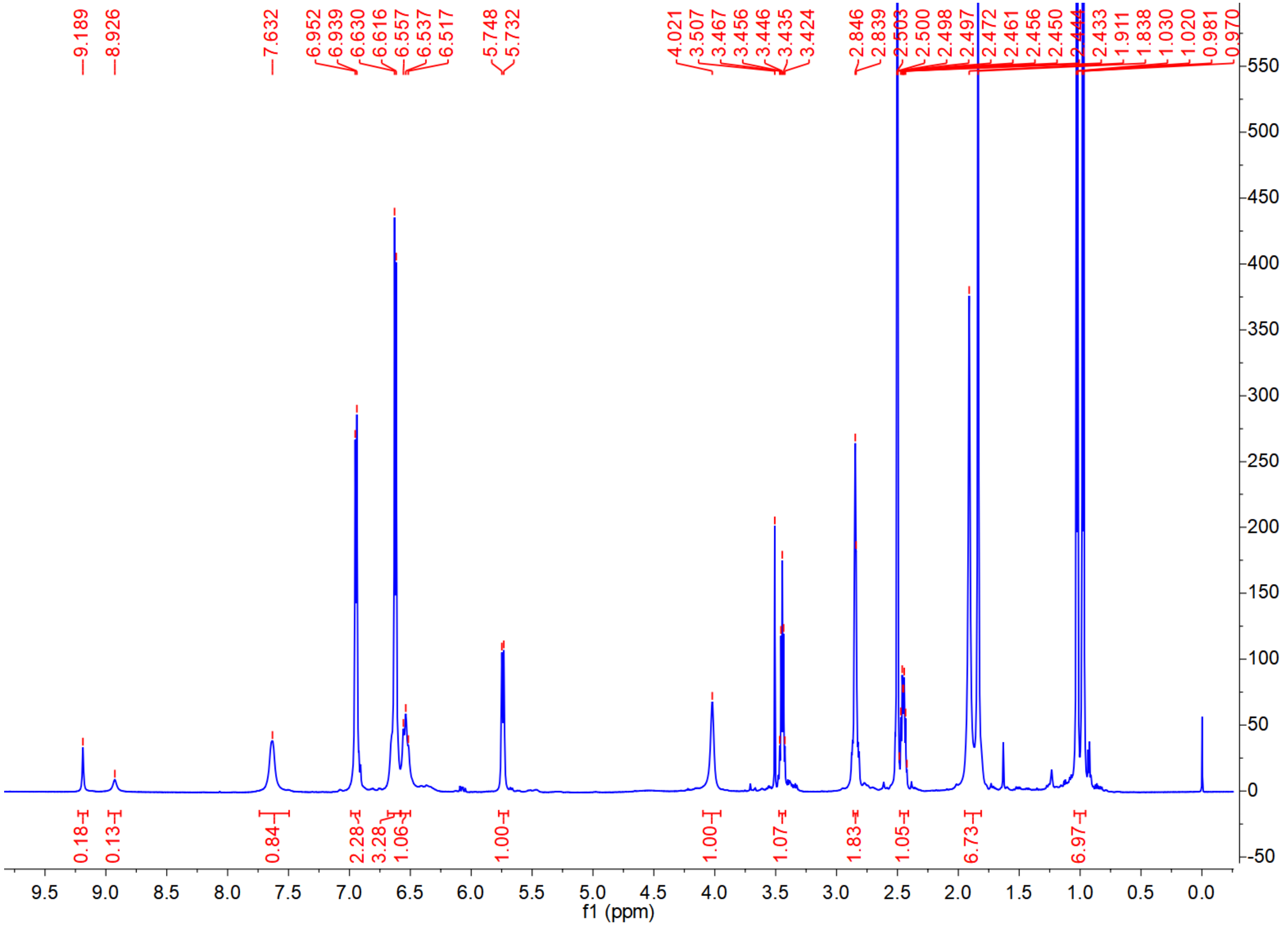

Figure S4. ^{13}C NMR spectrum of oxapyrrolidine A (**1**) in DMSO-d_6

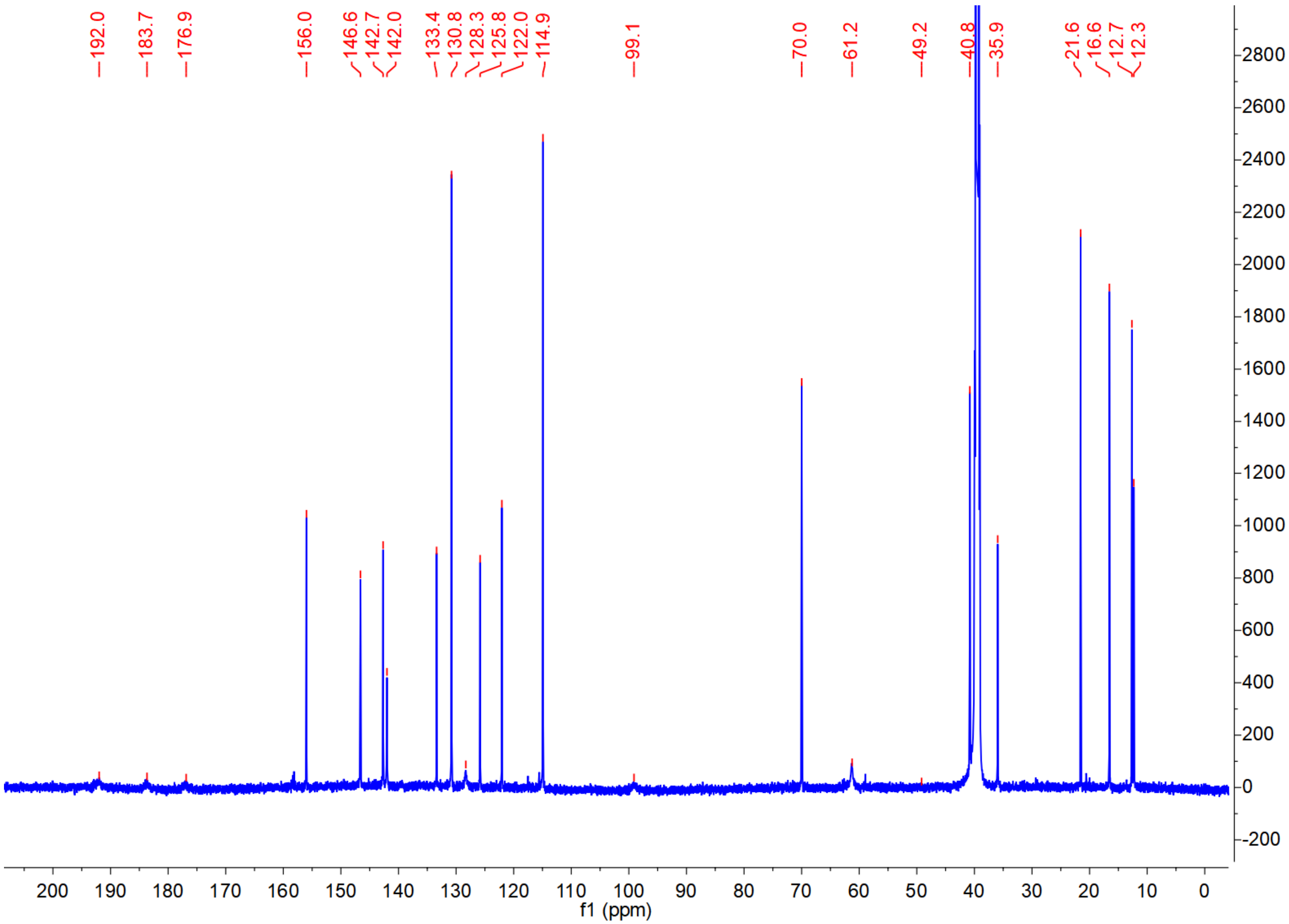

Figure S5. DEPT 135 spectrum of oxapyrrolidine A (**1**) in DMSO-d_6

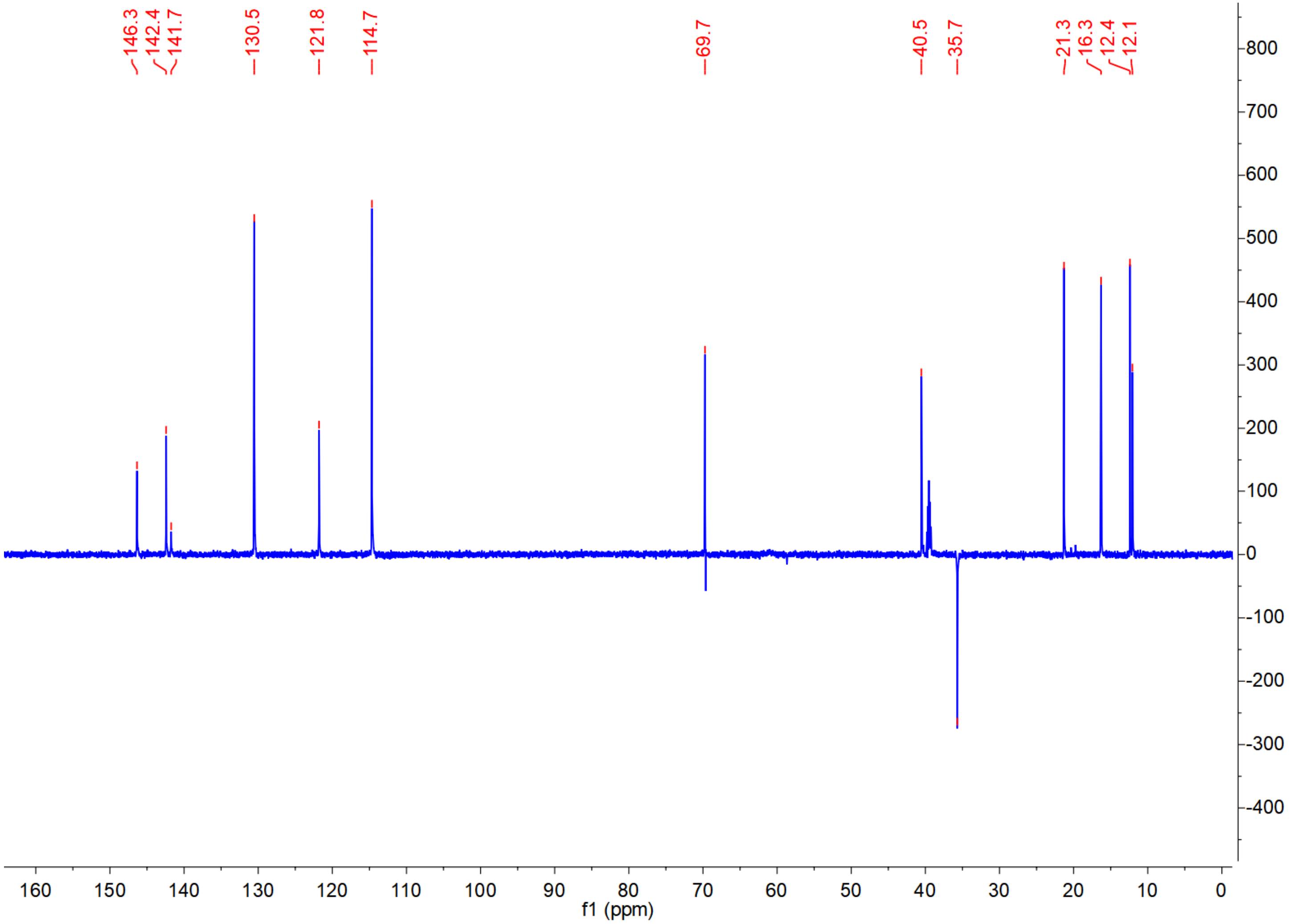

Figure S6. HSQC spectrum of oxapyrrolidine A (**1**) in DMSO-*d*6

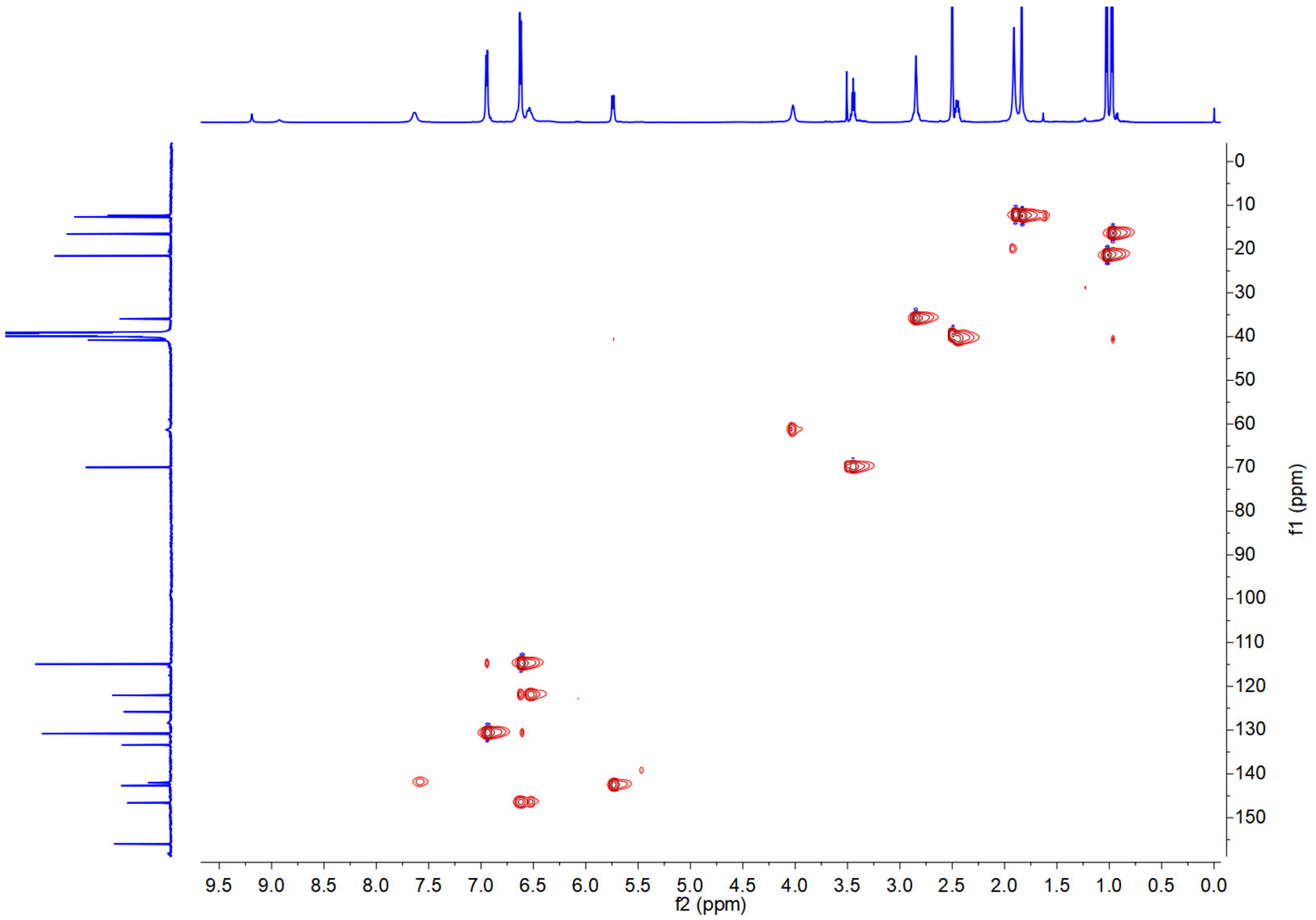

Figure S7. ^{1}H-^{1}H COSY spectrum of oxapyrrolidine A (**1**) in DMSO-d_6

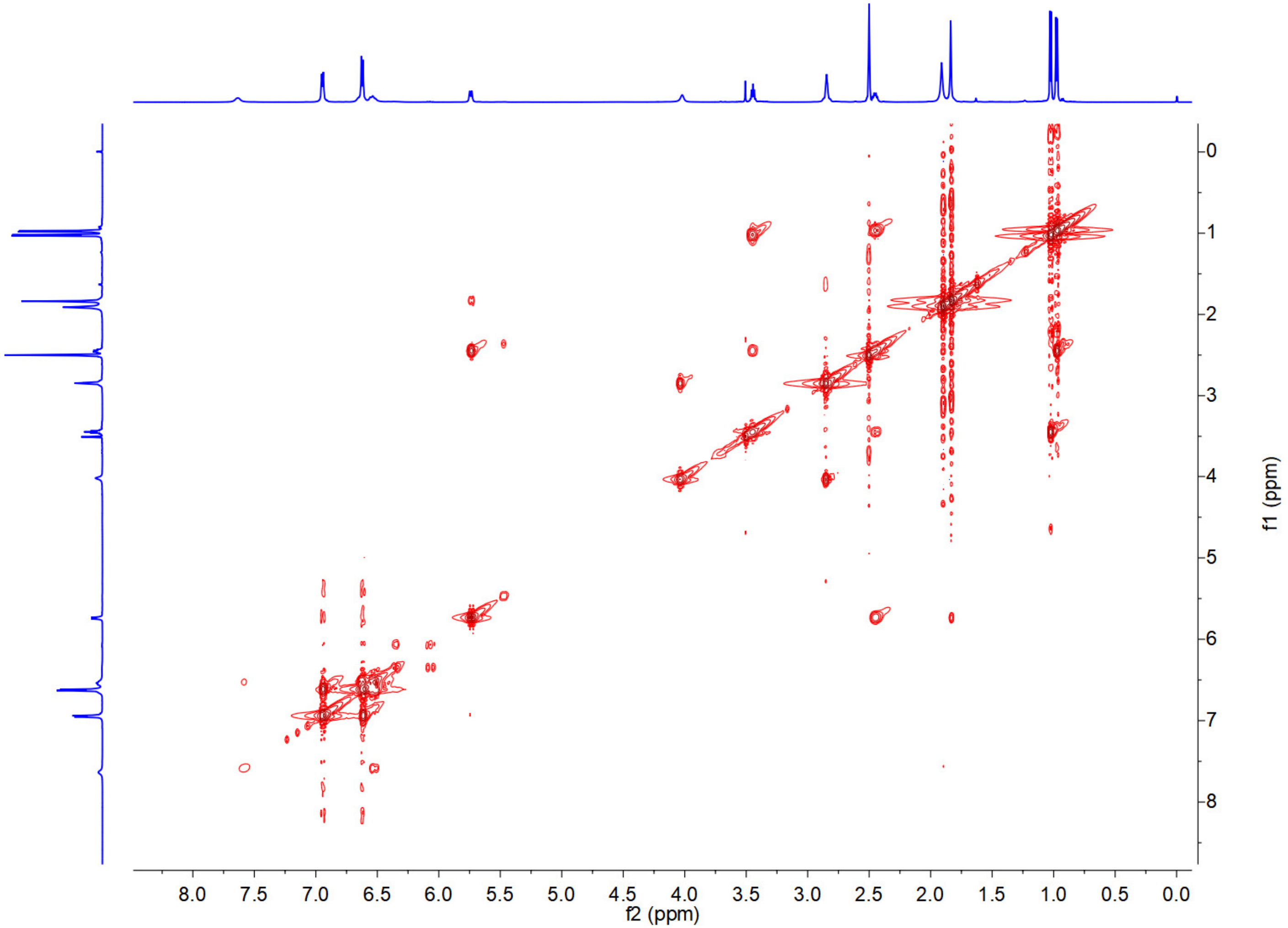

Figure S8. HMBC spectrum of oxapyrrolidine A (**1**) in DMSO-*d*6

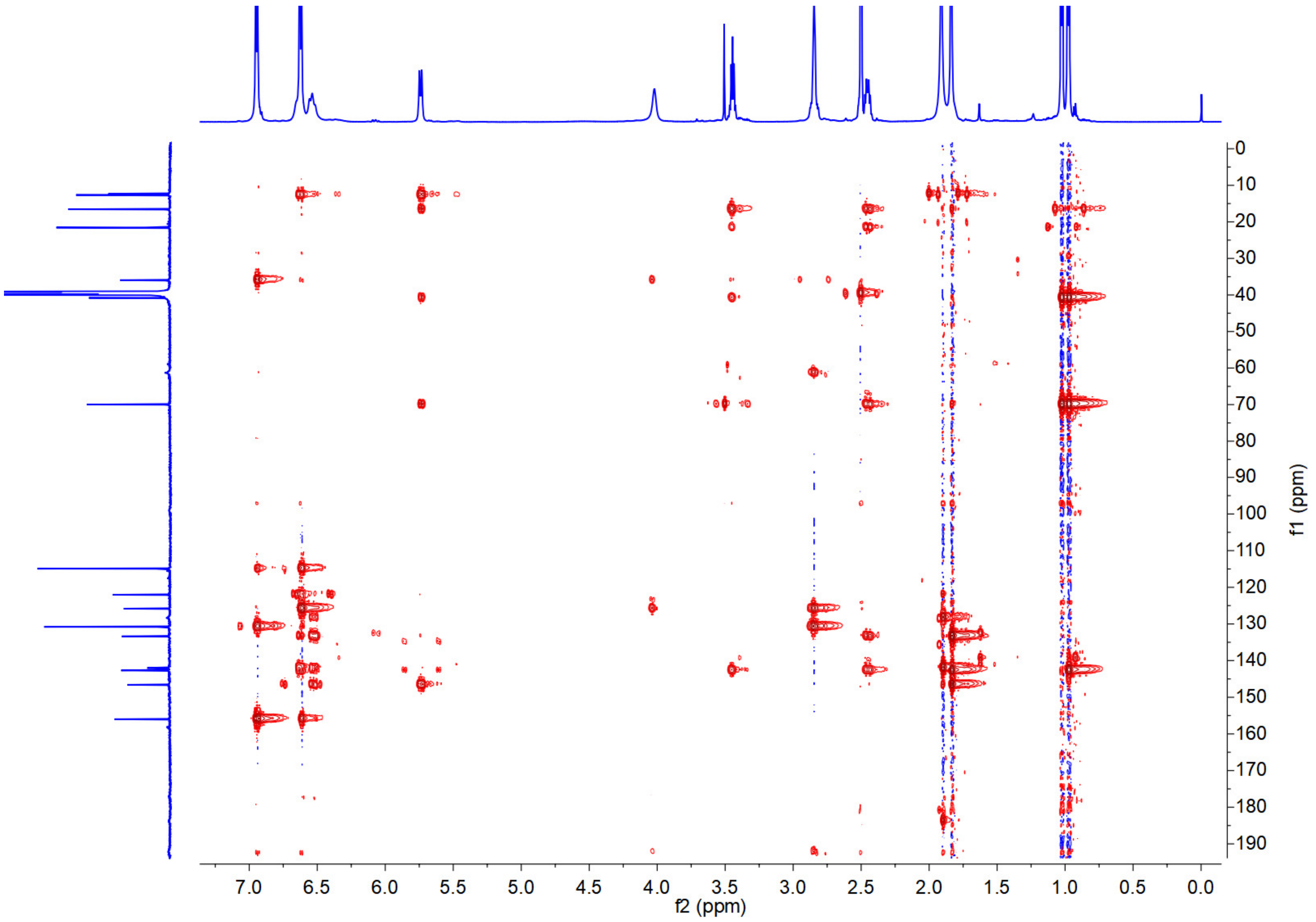

Figure S9. NOESY spectrum of oxapyrrolidine A (**1**) in DMSO-d_6

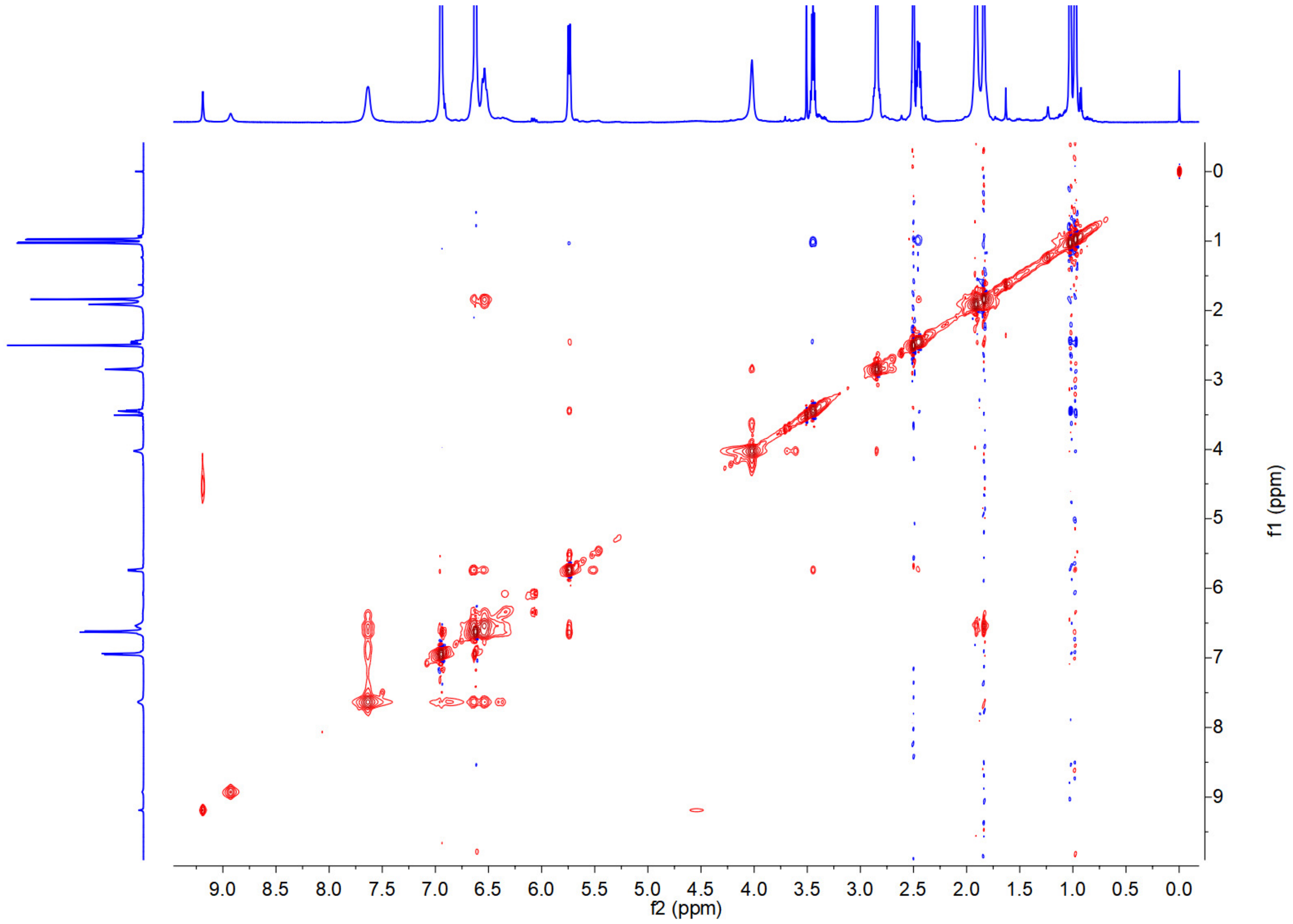

Figure S10. Key regions of ^{1}H spectrum of Mosher's ester of oxapyrrolidine A (**1**) in DMSO-d_6

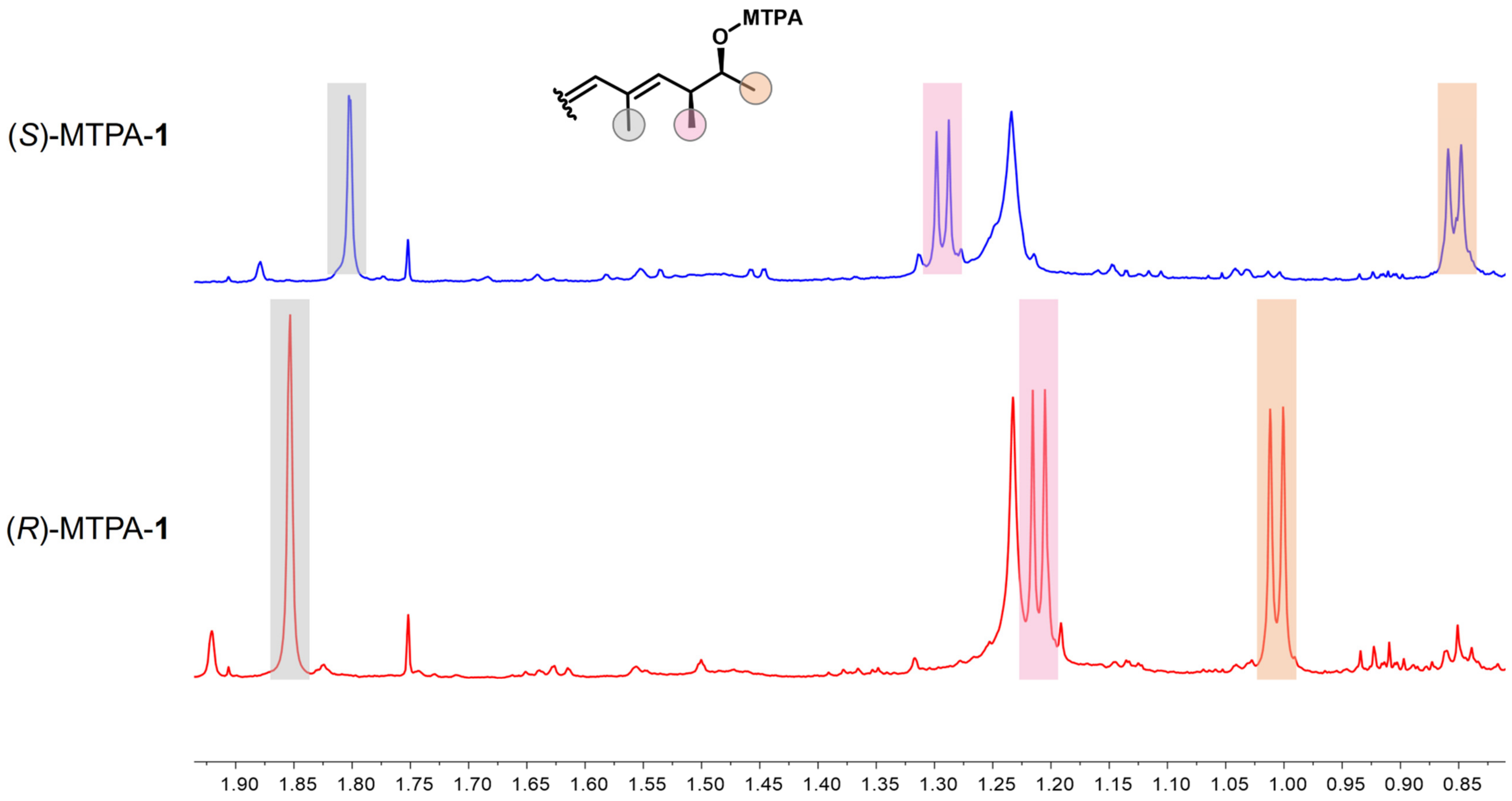

Figure S11. HECADE spectrum of oxapyrrolidine A (**1**) in DMSO-d_6

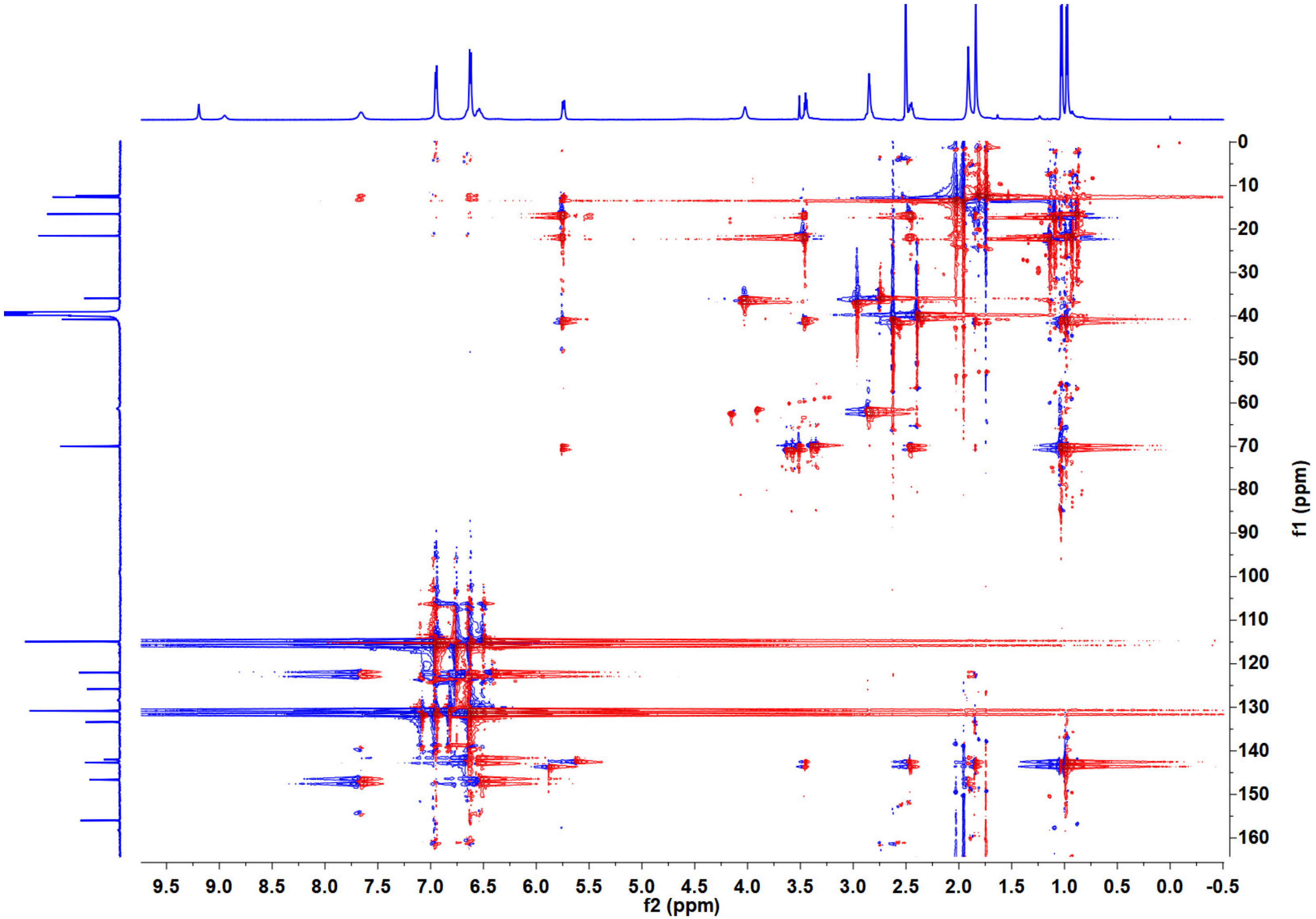

Figure S12. ECD spectrum of oxapyrrolidine A (**1**)

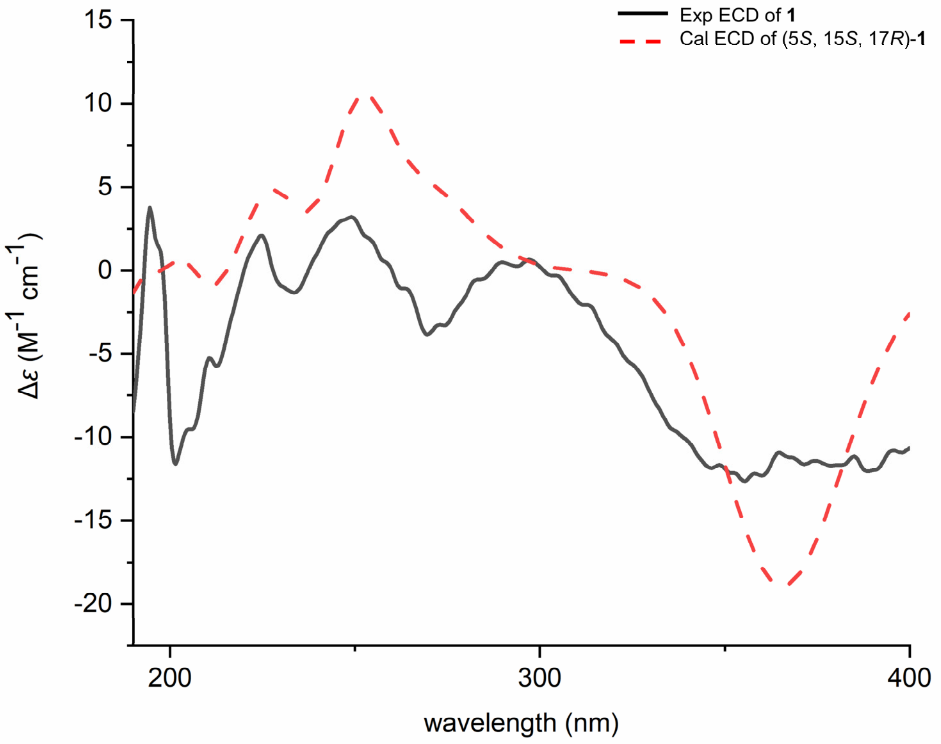

Figure S13. UV spectrum of oxapyrrolidine A (**1**)

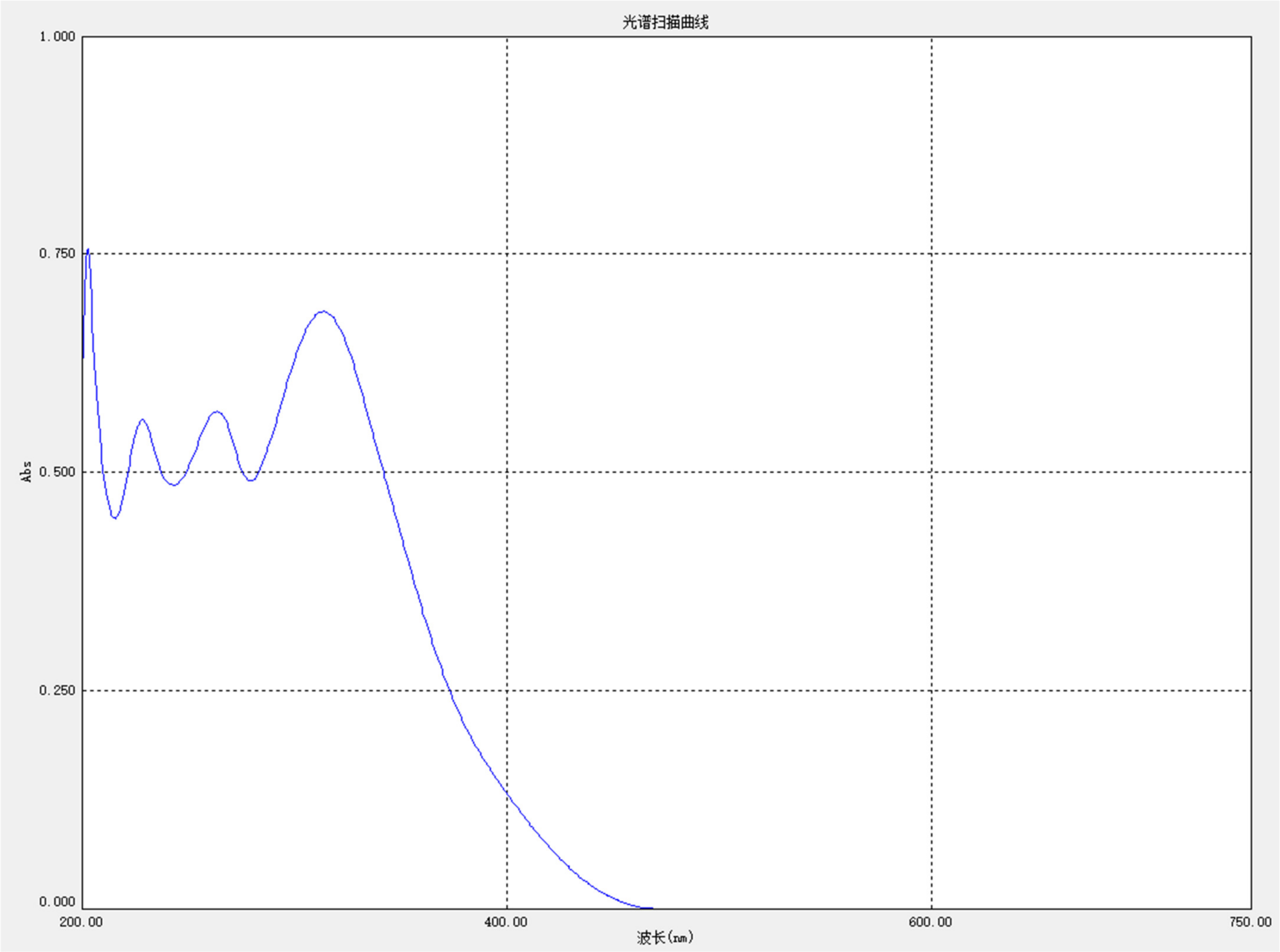

Figure S14. IR spectrum of oxapyrrolidine A (**1**)

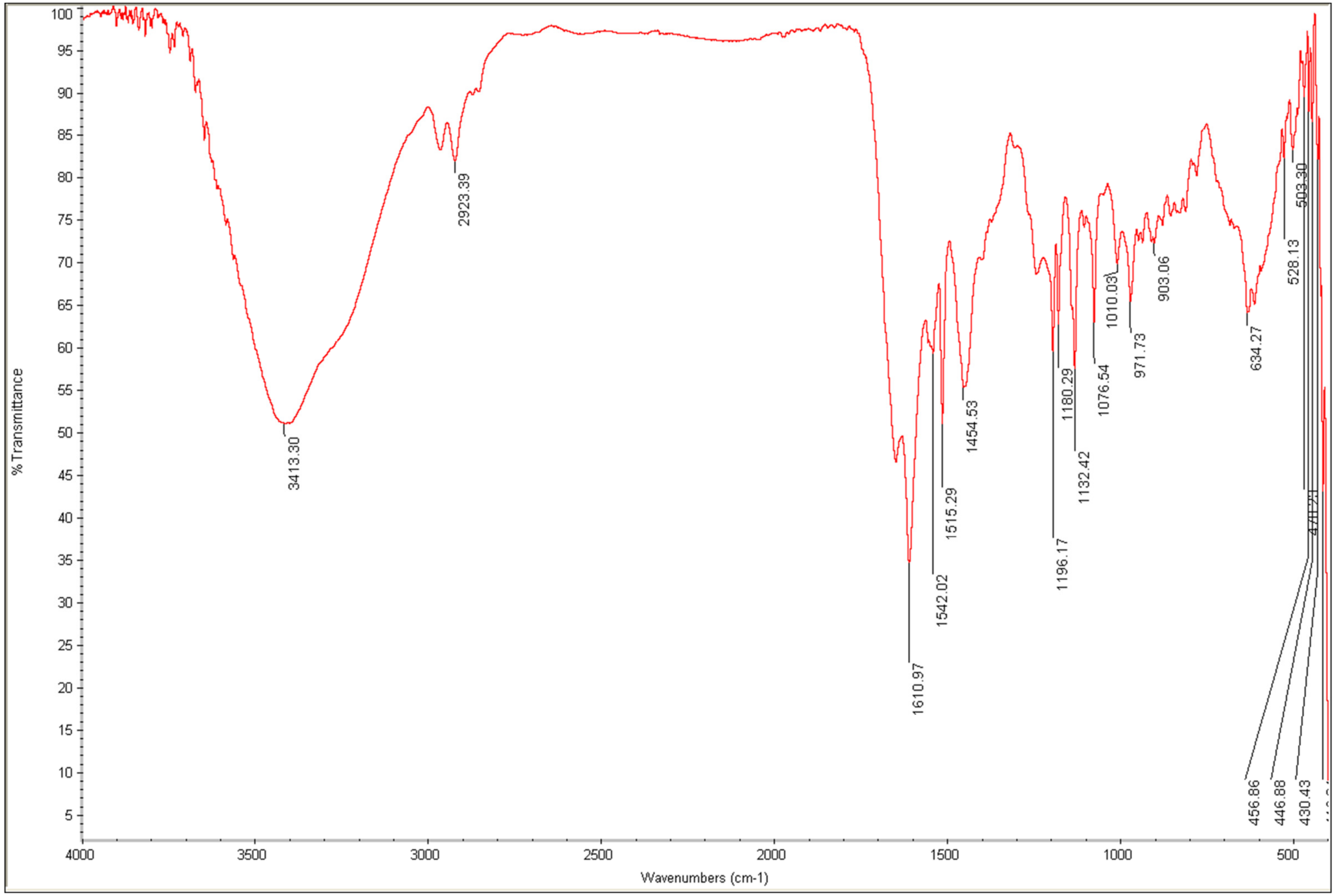

Figure S15. HR-ESI-MS spectrum of oxapyrrolidine B (**2**)

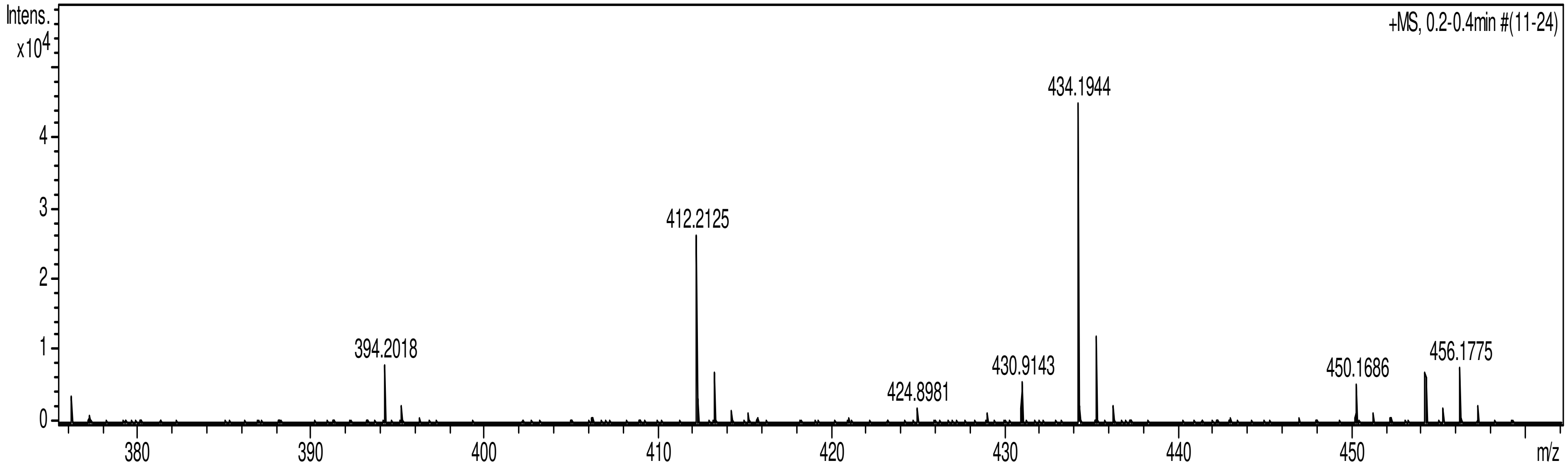

Figure S16. ^{1}H NMR spectrum of oxapyrrolidine B (**2**) in DMSO-d_6

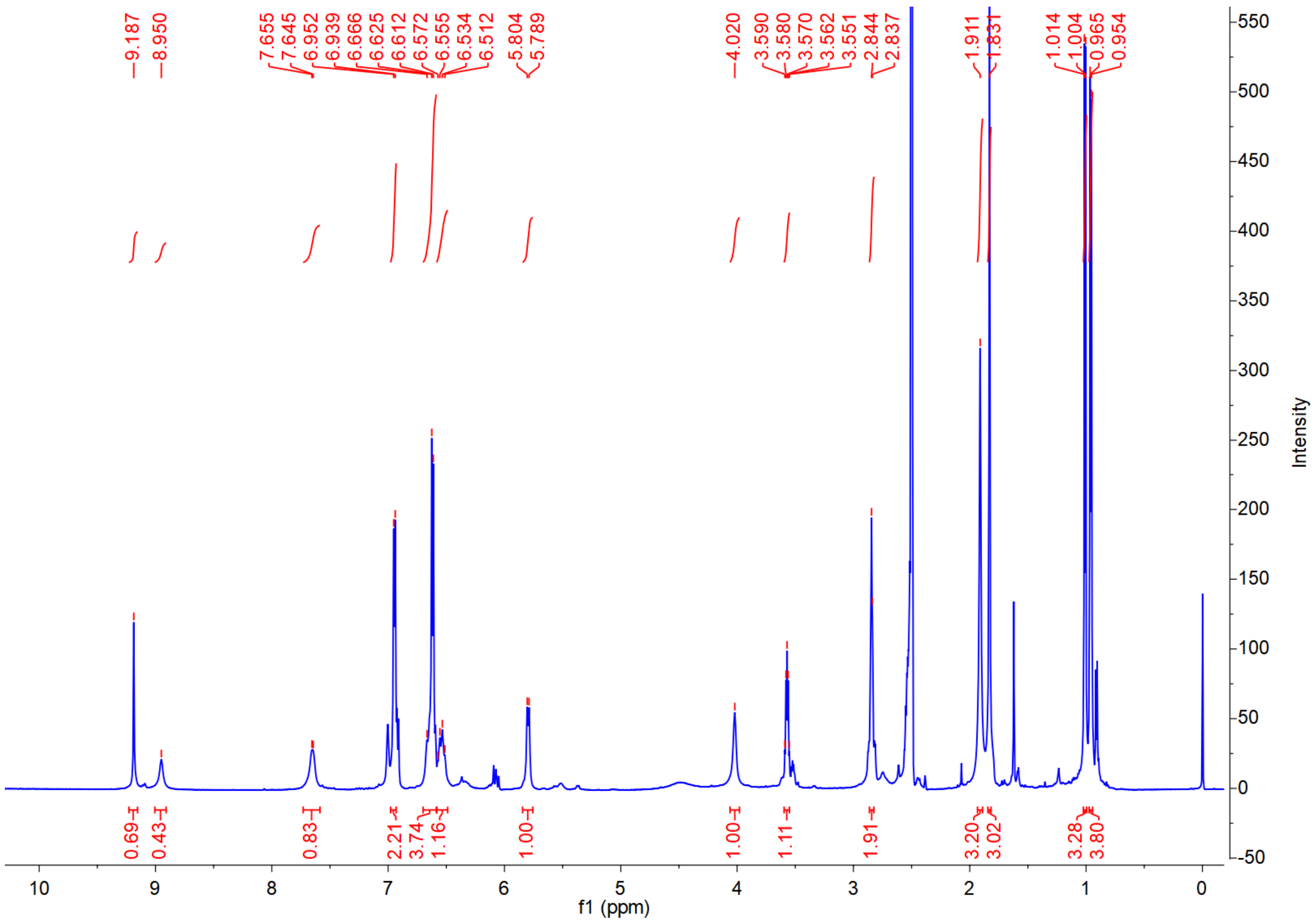

Figure S17. ^{13}C NMR spectrum of oxapyrrolidine B (**2**) in DMSO-d_6

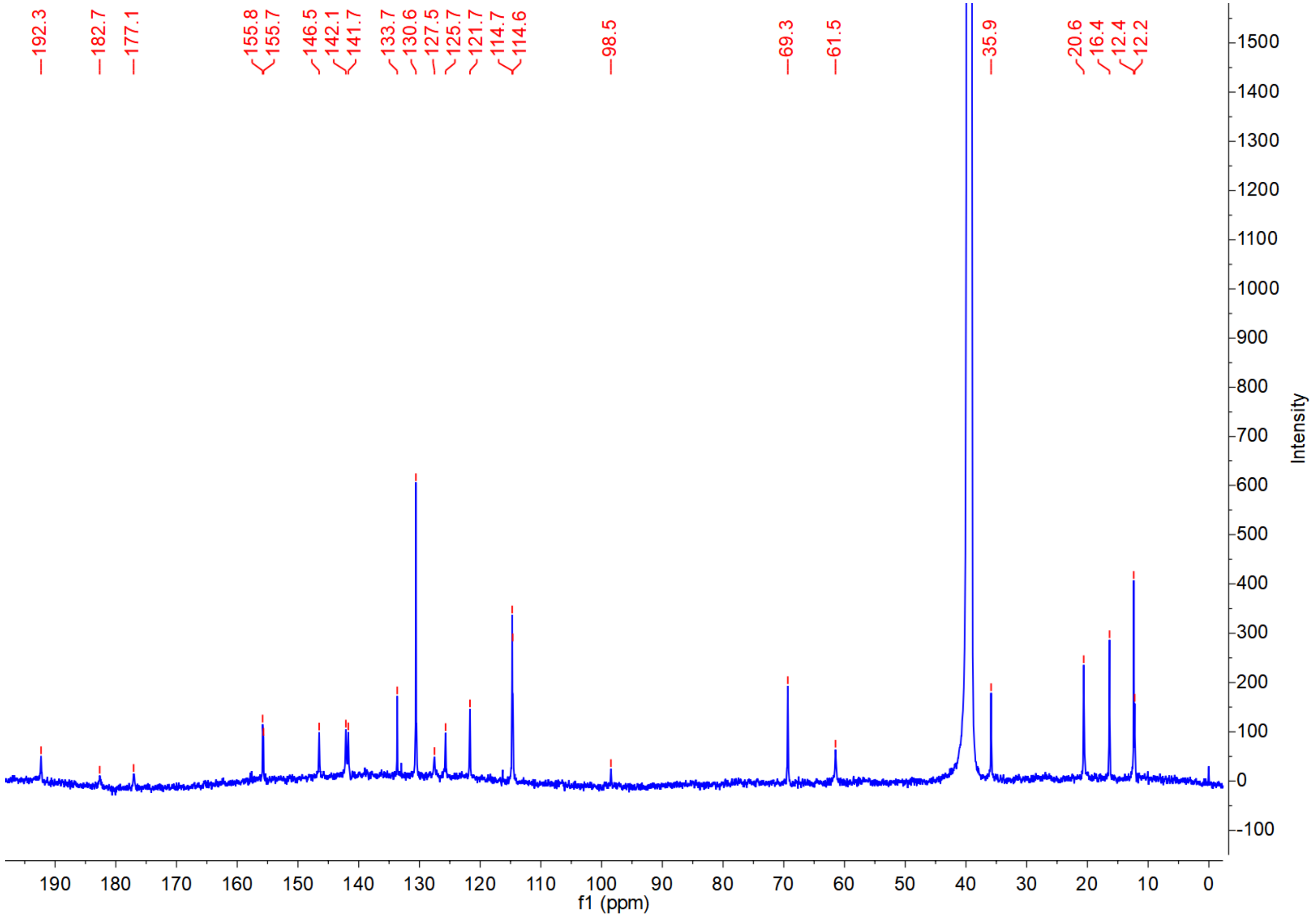

Figure S18. DEPT 135 spectrum of oxapyrrolidine B (**2**) in DMSO-d_6

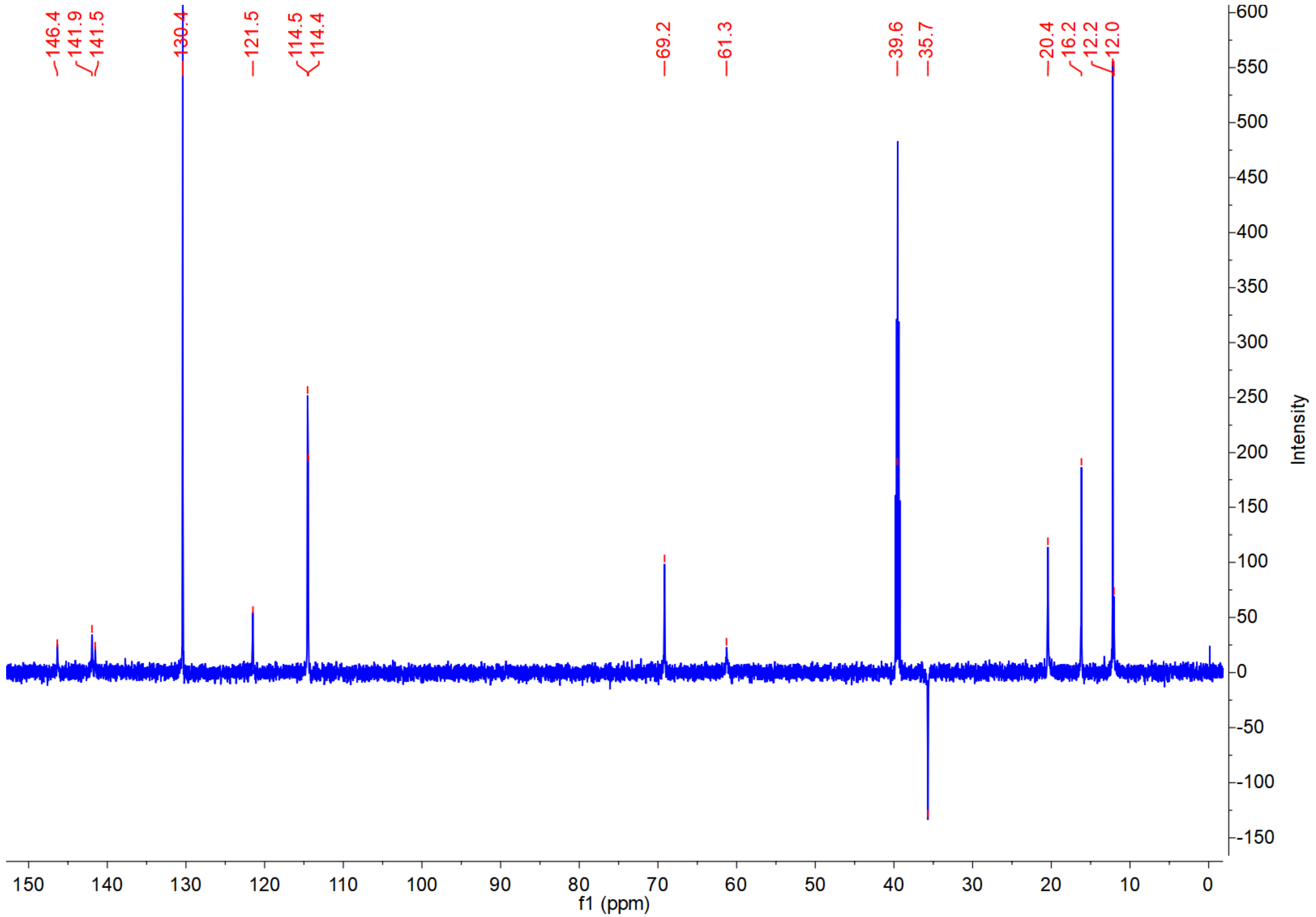

Figure S19. HSQC spectrum of oxapyrrolidine B (**2**) in DMSO-d_6

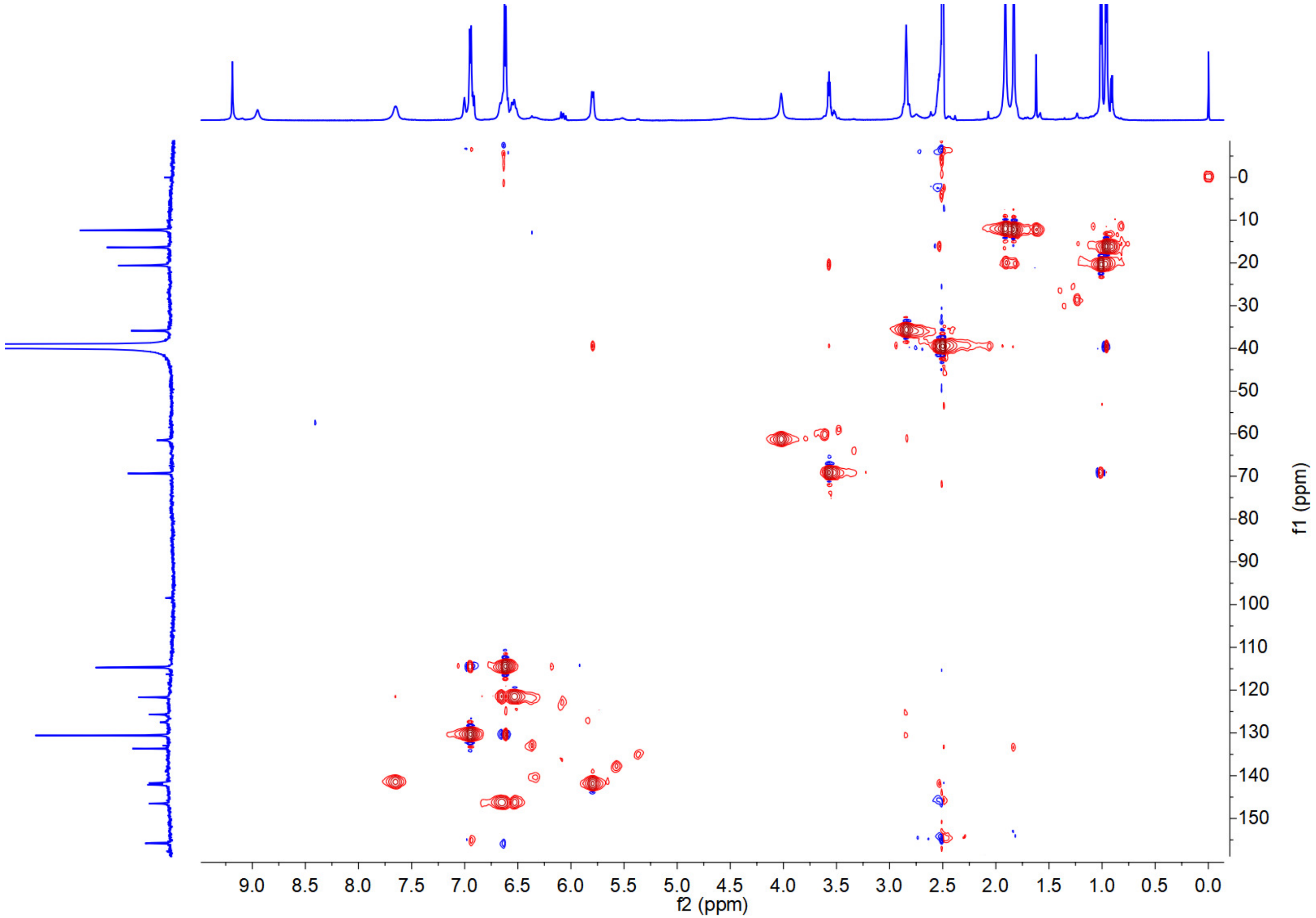

Figure S20. ^{1}H-^{1}H COSY spectrum of oxapyrrolidine B (**2**) in DMSO-*d*$_6$

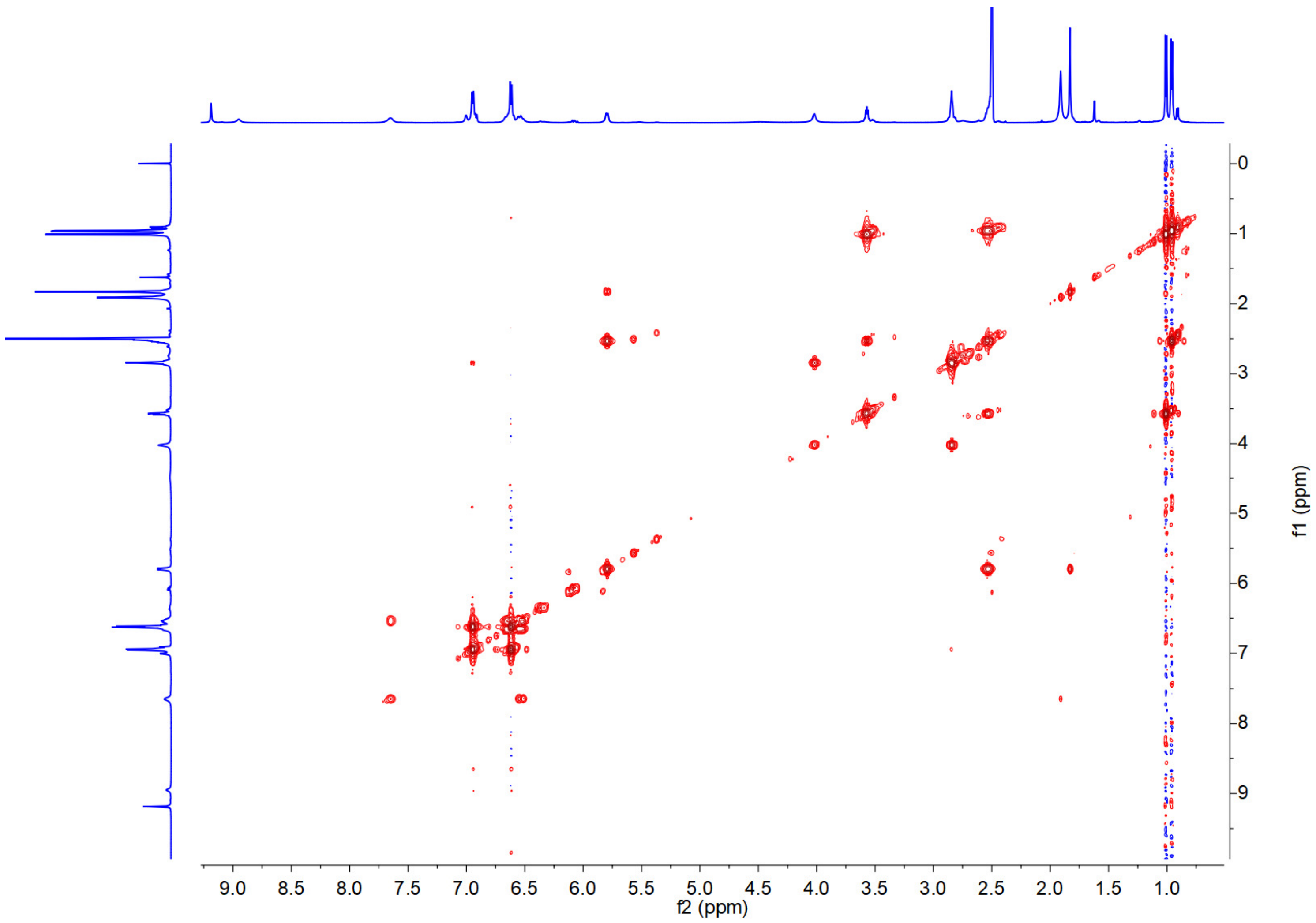

Figure S21. HMBC spectrum of oxapyrrolidine B (**2**) in DMSO-d_6

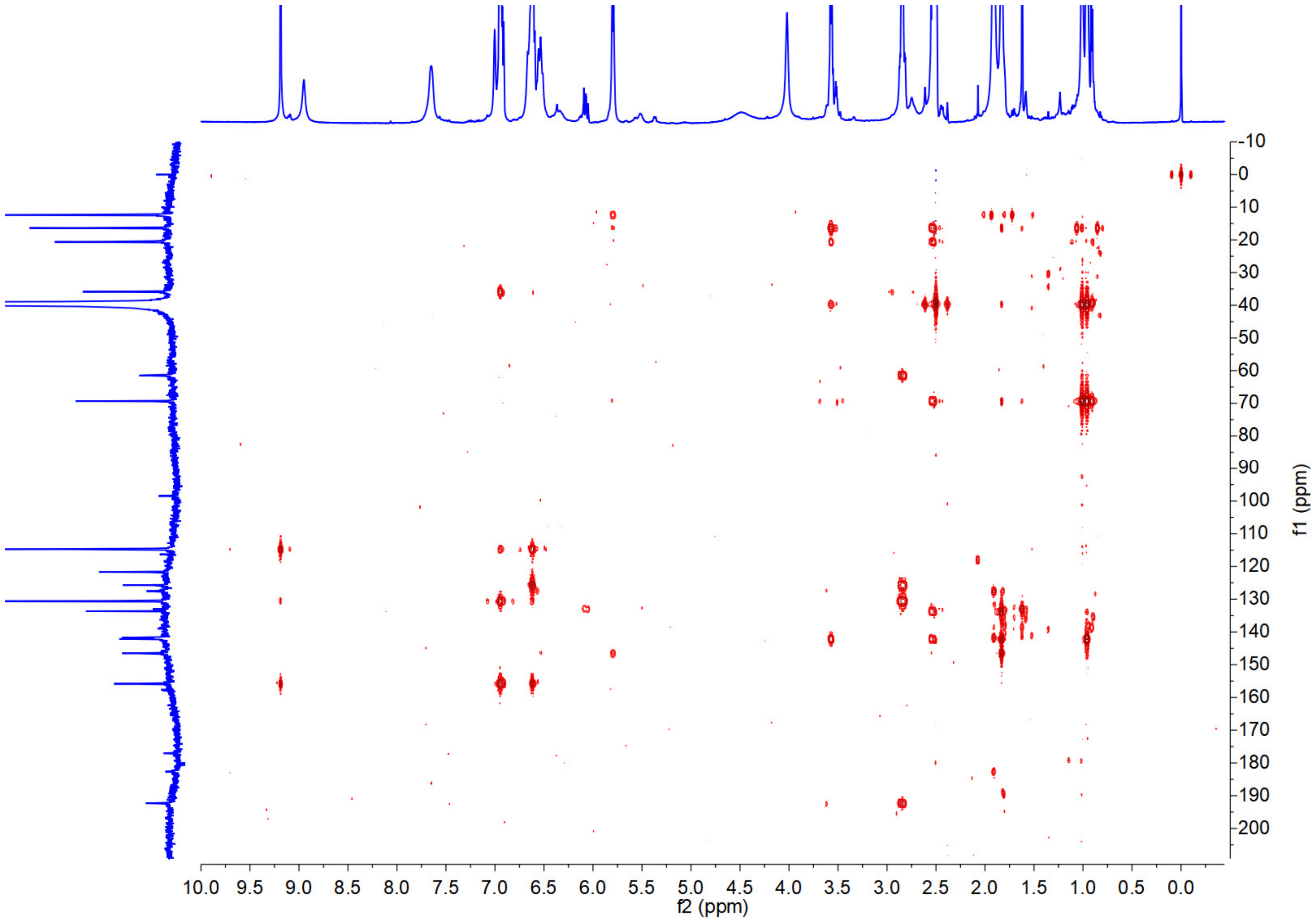

Figure S22. NOESY spectrum of oxapyrrolidine B (**2**) in DMSO-d_6

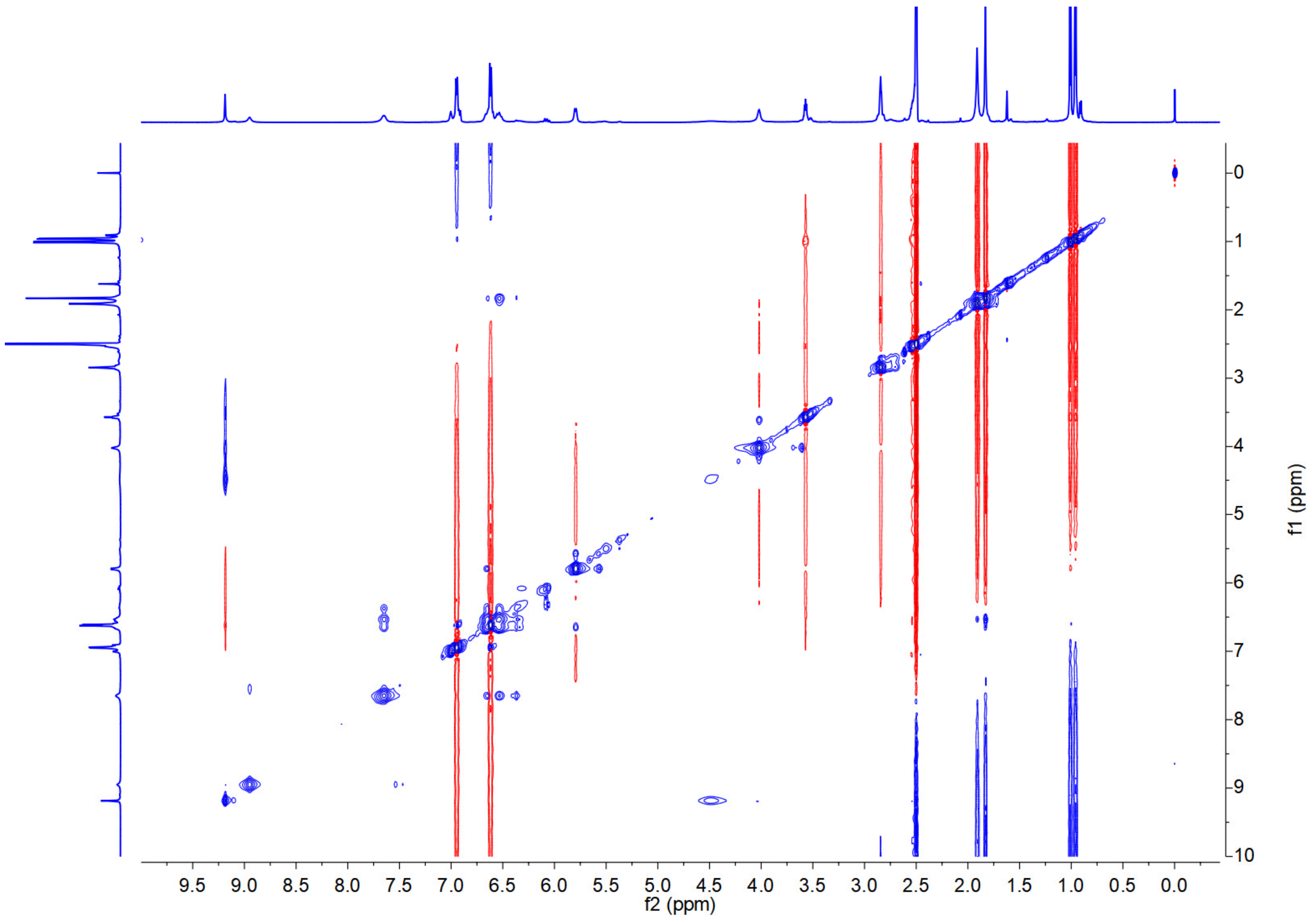

Figure S23. Key regions of ^{1}H spectrum of Mosher's ester of oxapyrrolidine B (**2**) in DMSO-d_6

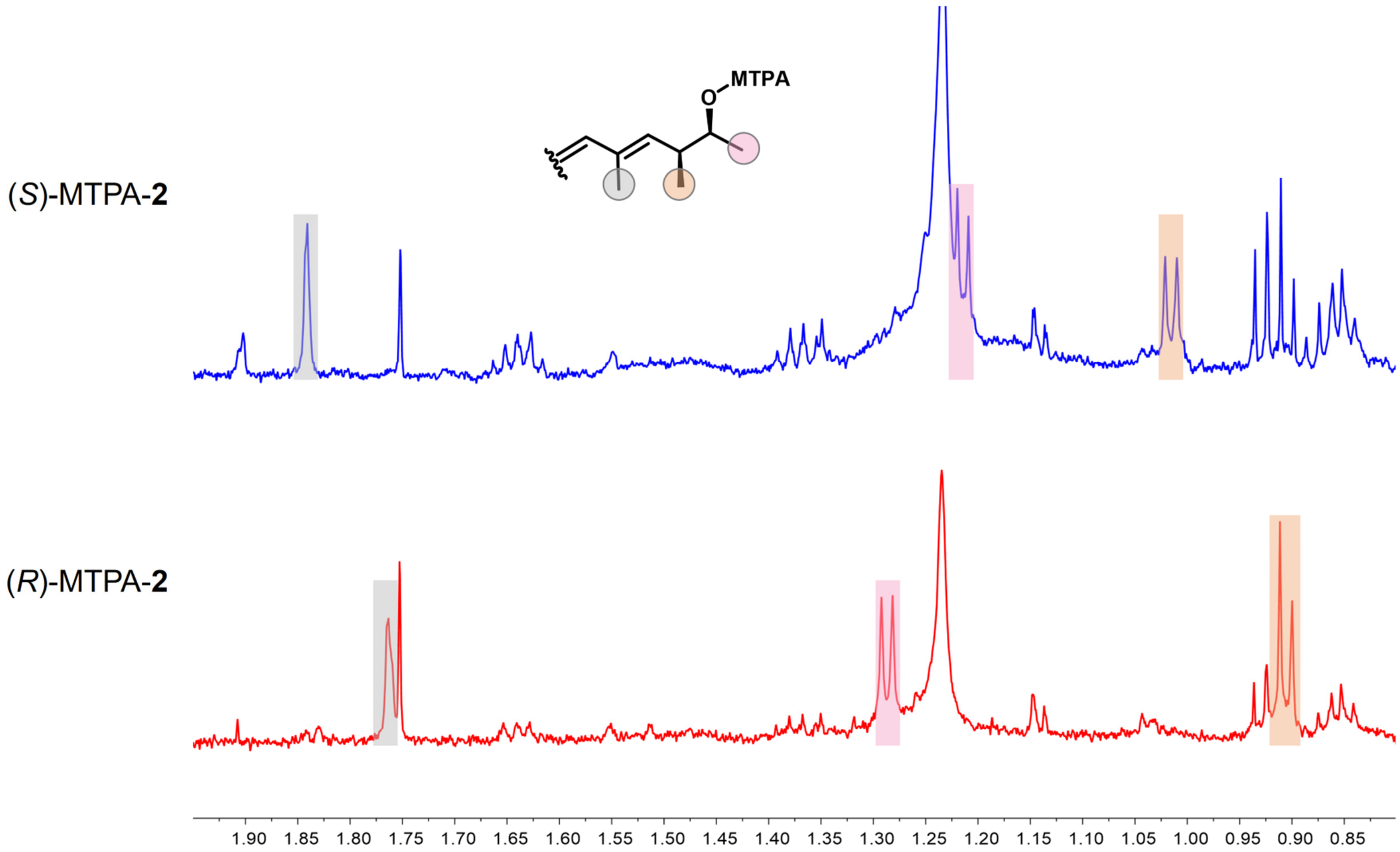

Figure S24. HECADE spectrum of oxapyrrolidine B (**2**) in DMSO-d_6

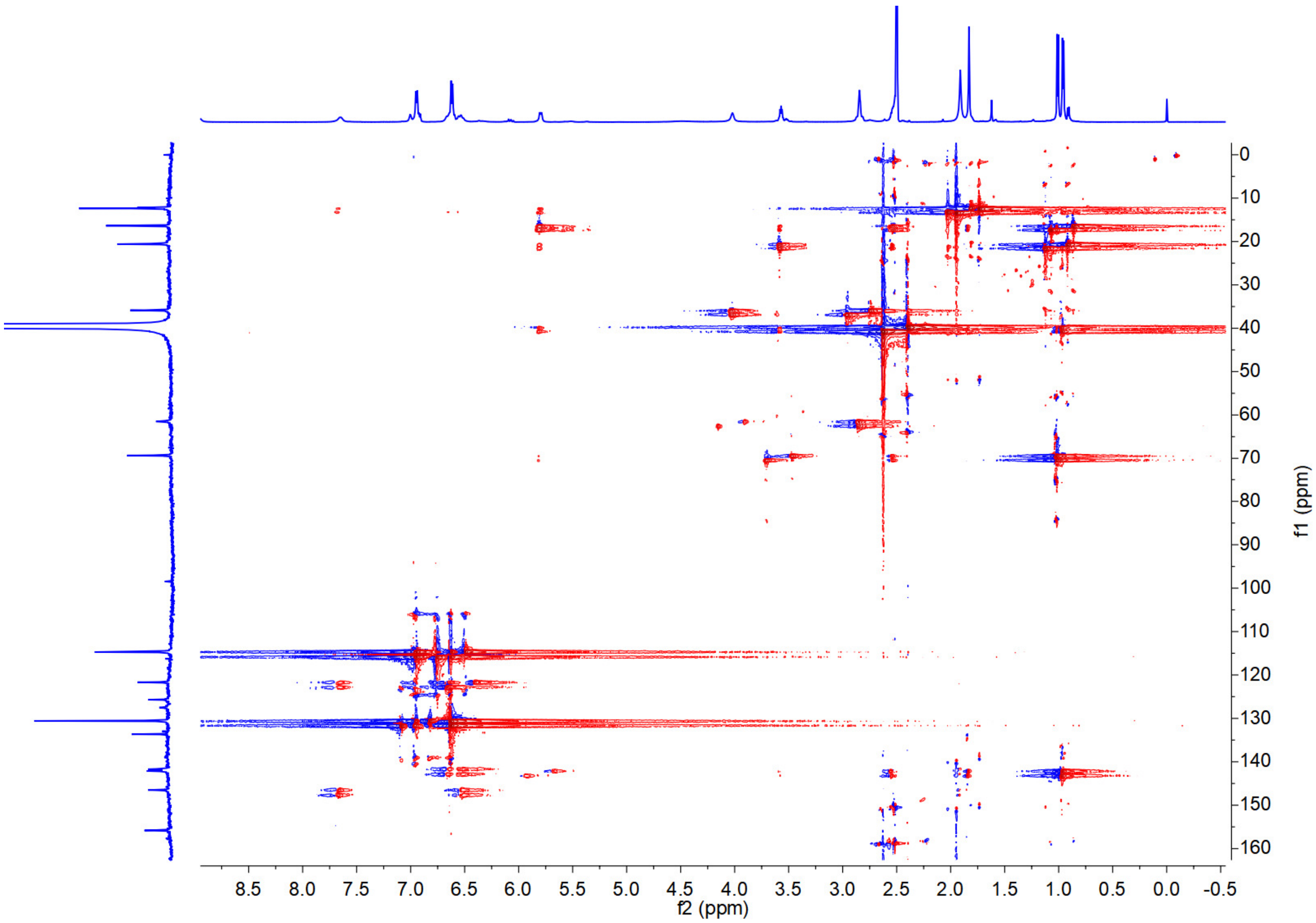

Figure S25. ECD spectrum of oxapyrrolidine B (**2**)

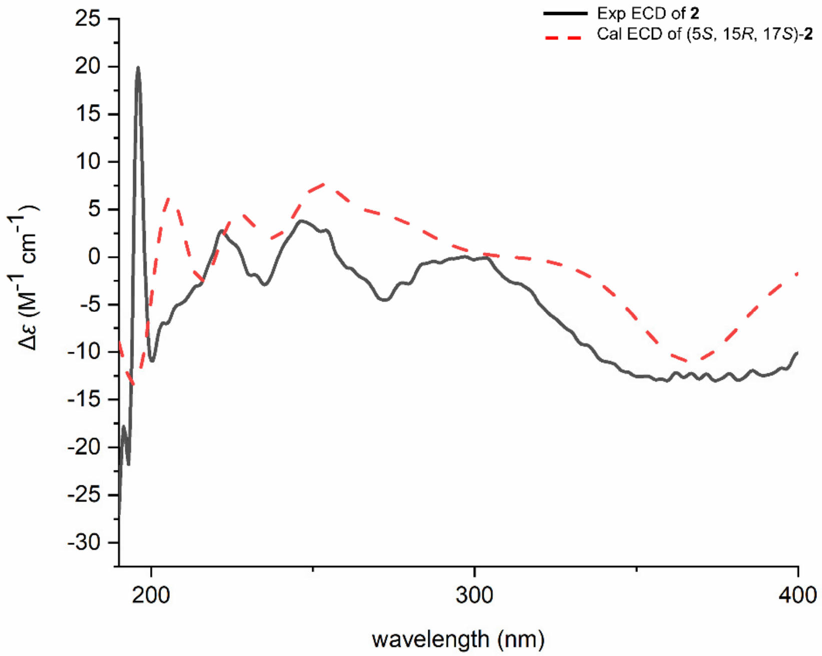

Figure S26. UV spectrum of oxapyrrolidine B (**2**)

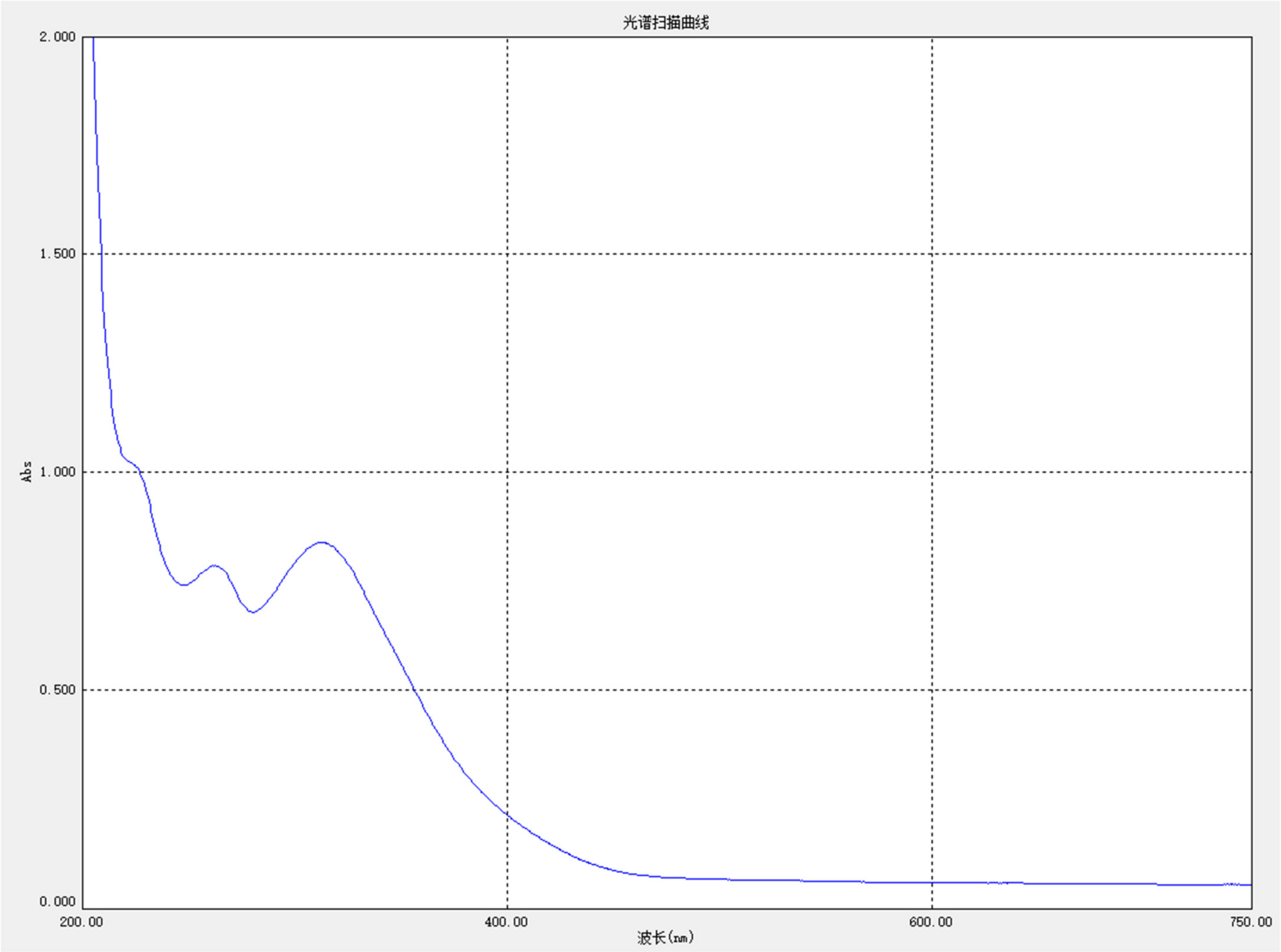

Figure S27. IR spectrum of oxapyrrolidine B (**2**)

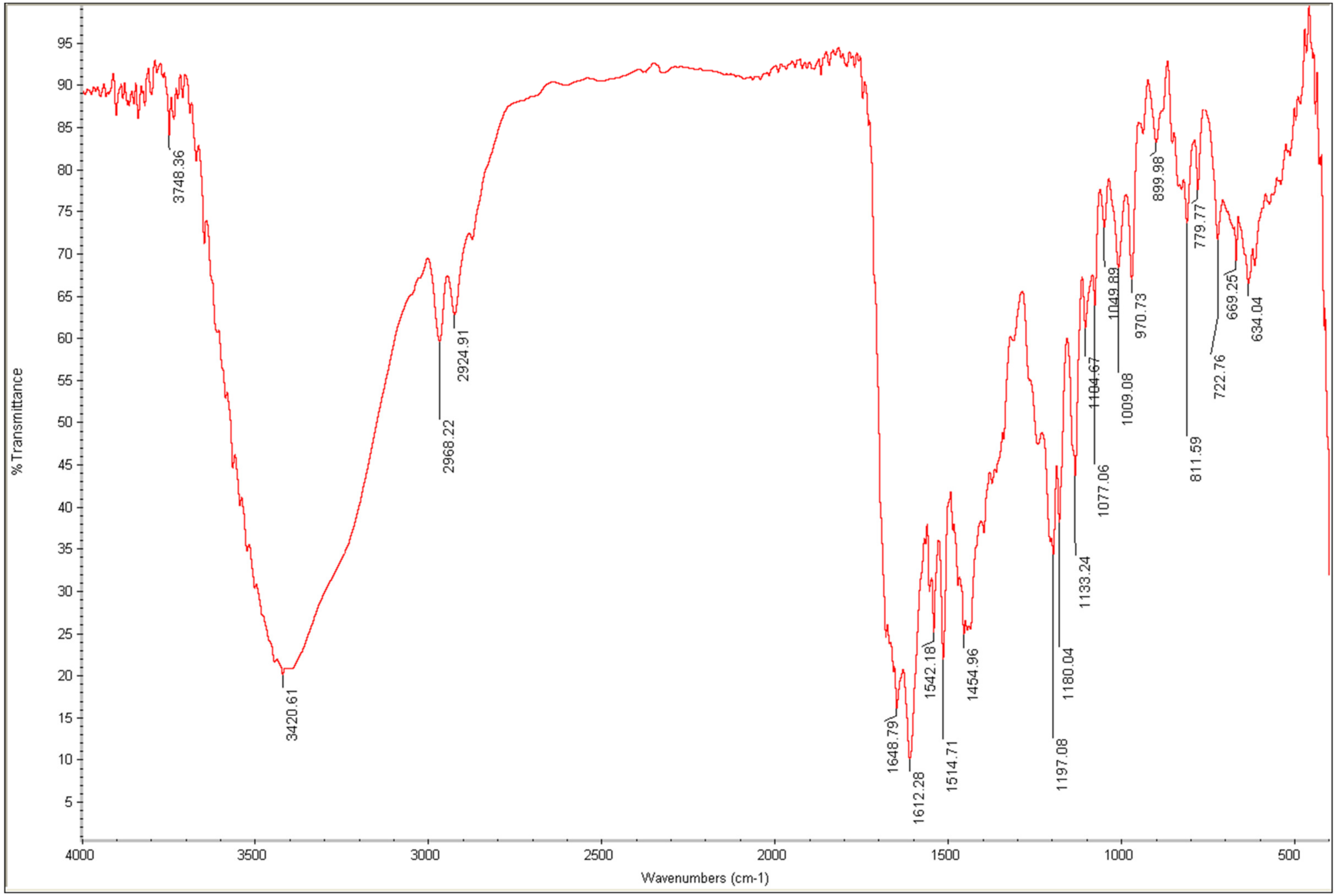

Figure S28. *J*-based analysis including all conformer pairs that produce a medium $^3J_{(H14\text{-}H15)}$ [1]

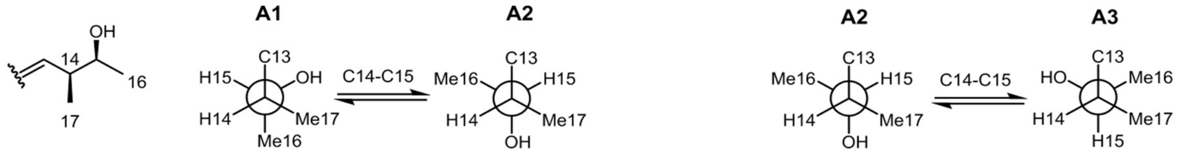

	A1	A2		A2	A3		**16** (Hz)	**17** (Hz)	
$^3J_{(H14, H15)}$	Small (0-4 Hz)	Large (6-8 Hz)	Medium (4-6 Hz)	Large (8-12 Hz)	Small (0-4 Hz)	Medium (4-6 Hz)	6.2	6.2	Medium (4-6 Hz)
$^3J_{(Me16, H14)}$	Small (1-3 Hz)	Small (1-3 Hz)	Small (1-3 Hz)	Small (1-3 Hz)	Large (6-8 Hz)	Medium (3-6 Hz)	2.7	1.6	Small (1-3 Hz)
$^3J_{(C13, H15)}$	Small (1-3 Hz)	Small (1-3 Hz)	Small (1-3 Hz)	Small (1-3 Hz)	Large (6-8 Hz)	Medium (3-6 Hz)	2.8	2.3	Small (1-3 Hz)
$^3J_{(Me17, H15)}$	Large (6-8 Hz)	Medium (3-6 Hz)	Medium (3-6 Hz)	Small (1-3 Hz)	Small (1-3 Hz)	Small (1-3 Hz)	3.9	3.4	Medium (3-6 Hz)
$^2J_{(C15, H14)}$	Small (0-2 Hz)	Large (5-7 Hz)	Medium (2-5 Hz)	Large (5-7 Hz)	Large (5-7 Hz)	Large (5-7 Hz)	4.5	4.2	Medium (2-5 Hz)

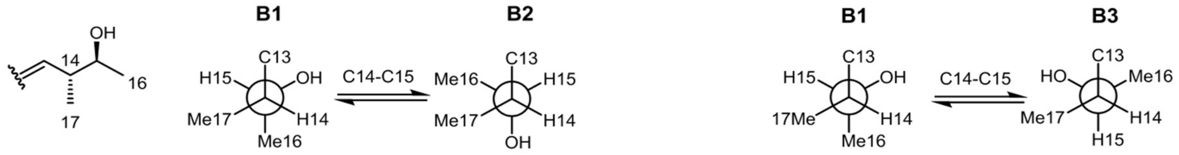

	B1	B2		B1	B3		**16** (Hz)	**17** (Hz)	
$^3J_{(H14, H15)}$	Large (8-12 Hz)	Small (0-4 Hz)	Medium (4-6 Hz)	Large (8-12 Hz)	Small (0-4 Hz)	Medium (4-6 Hz)	6.2	6.2	Medium (4-6 Hz)
$^3J_{(Me16, H14)}$	Small (1-3 Hz)	Large (6-8 Hz)	Medium (3-6 Hz)	Small (1-3 Hz)	Small (1-3 Hz)	Small (1-3 Hz)	2.7	1.6	Small (1-3 Hz)
$^3J_{(C13, H15)}$	Small (1-3 Hz)	Small (1-3 Hz)	Small (1-3 Hz)	Small (1-3 Hz)	Large (6-8 Hz)	Medium (3-6 Hz)	2.8	2.3	Small (1-3 Hz)
$^3J_{(Me17, H15)}$	Small (1-3 Hz)	Large (6-8 Hz)	Medium (3-6 Hz)	Small (1-3 Hz)	Small (1-3 Hz)	Small (1-3 Hz)	3.9	3.4	Medium (3-6 Hz)
$^2J_{(C15, H14)}$	Large (5-7 Hz)	Large (5-7 Hz)	Large (5-7 Hz)	Large (5-7 Hz)	Small (0-2 Hz)	Medium (2-5 Hz)	4.5	4.2	Medium (2-5 Hz)

1. Neuhaus, G.F.; Adpressa, D.A.; Bruhn, T.; Loesgen, S. Polyketides from marine-derived *Aspergillus porosus*: challenges and opportunities for determining absolute configuration. *J. Nat. Prod.* **2019**, *82*, 2780-2789.

Figure S29. HPLC profiles of extracts from wild-type and mutants.

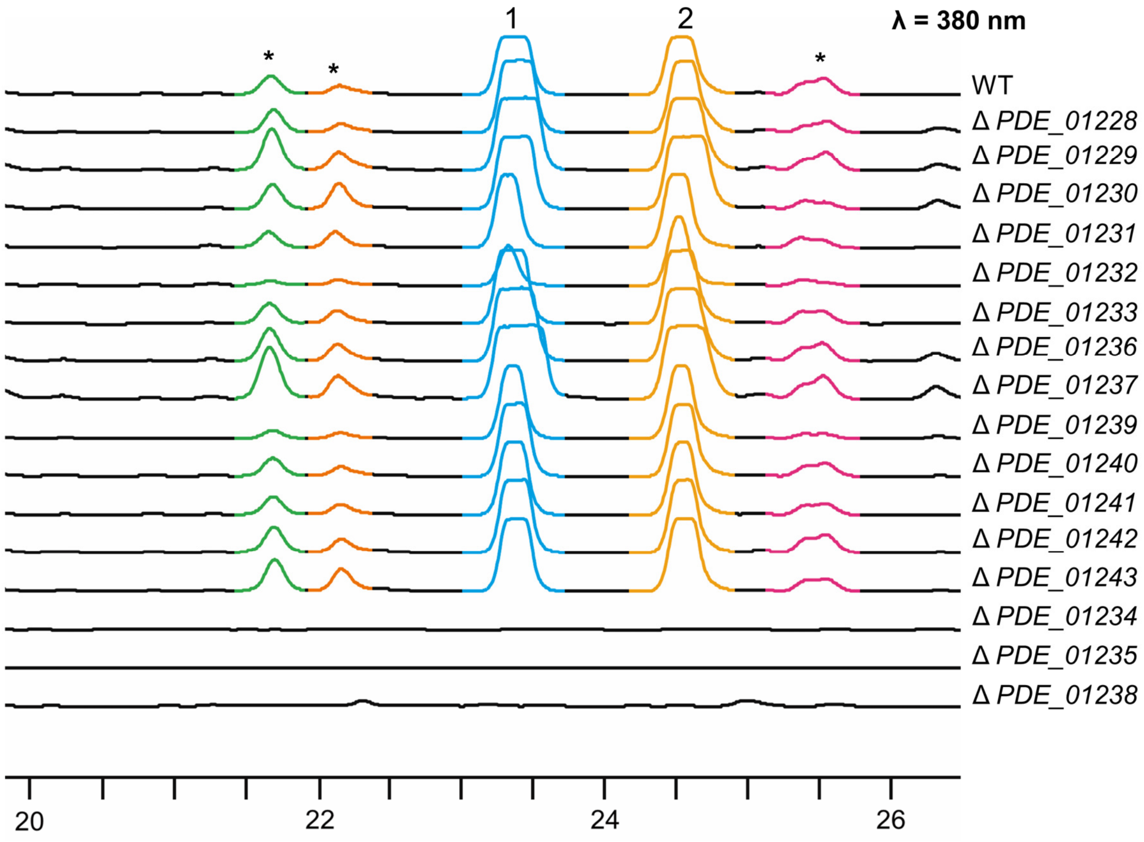

The asterisk-marked peaks represent the analogs of **1** and **2**.

Figure S30. Extracted ion chromatograms of extracts from wild-type and mutants

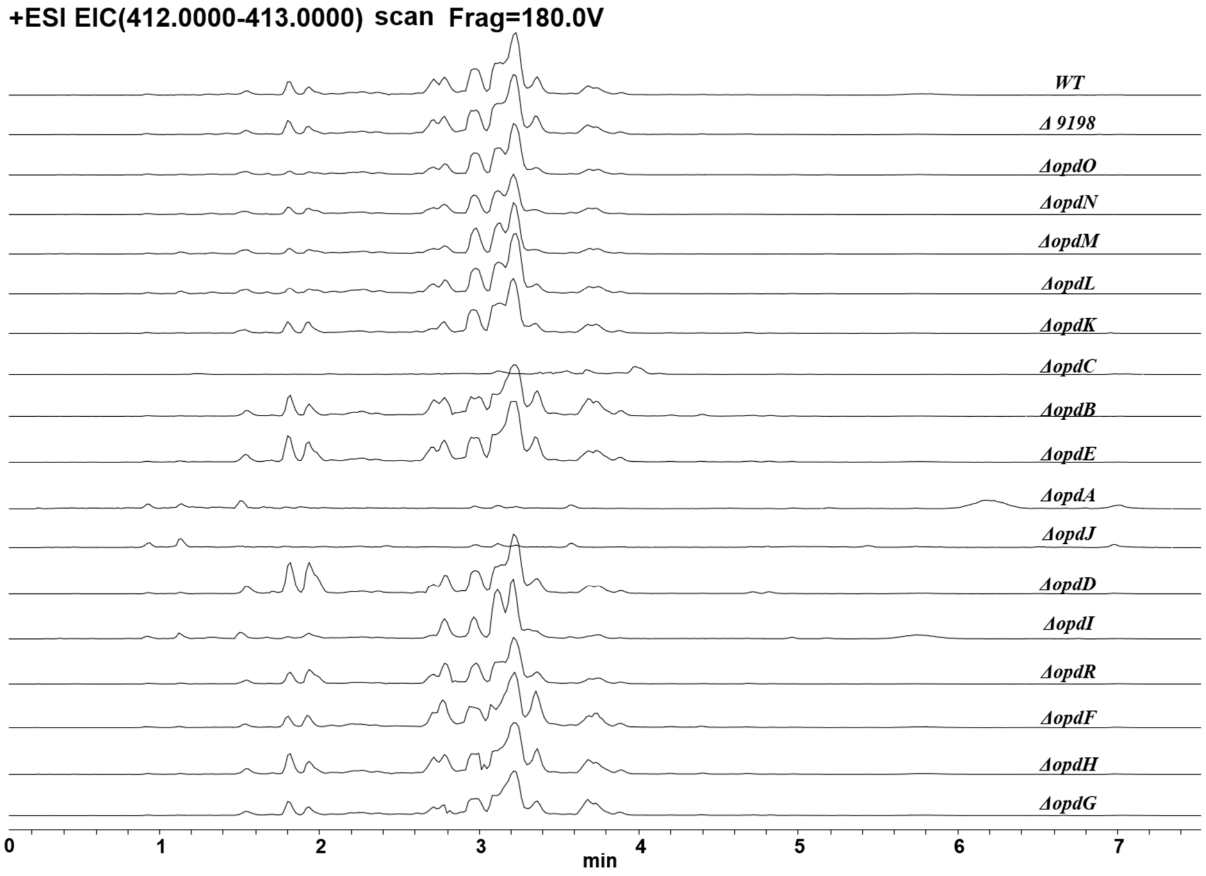

Figure S31. Stereochemical course of fungal reduced polyketides.

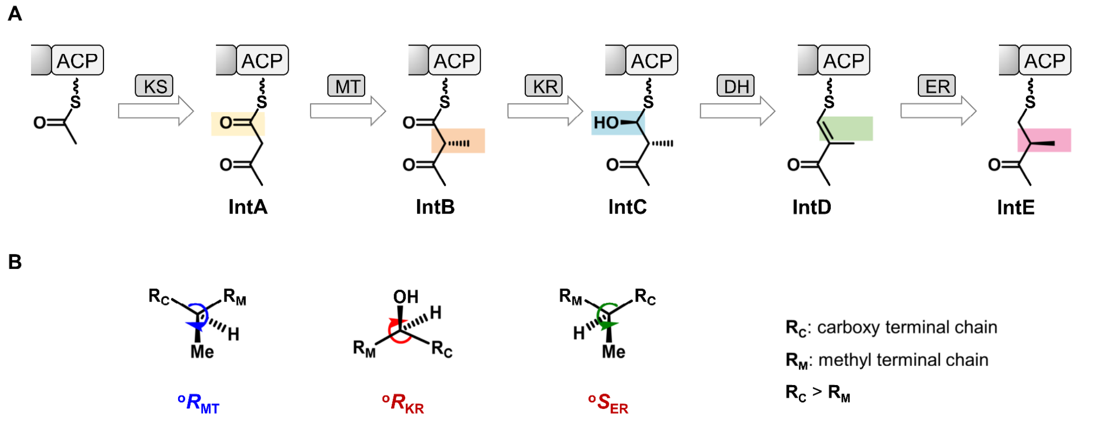

(A) absolute configurations of stereogenic centers in fungal reduced polyketides. (B) Newly defined °*R*/°*S* model. The newly defined °*R*/°*S* model focuses on the direction of the polyketide chain elongation and does not consider the absolute configurations of other nearby substituents. In the stereochemical model, the author defined that a carboxy-terminal chain (R_C) has a higher priority than a methyl terminal chain (R_M).

Oikawa and coworkers have proposed a stereochemical rule to explain the absolute configurations of fungal reduced polyketides [1]. During the polyketide elongation, an °R_{MT}-methyl group was introduced into IntA to form IntB by the methyltransferase (MT) domain. Then, the ketoreductase (KR) domains reduced IntB to give IntC with an °R_{KR}-hydroxy group. Subsequently, dehydratase (DH) domains catalyzed *E*-olefin formation to afford IntD. Finally, enoyl was reduced by enoyl reductases (ER), giving IntE with an °S_{ER}-methyl group. This stereochemical rule can explain the absolute configurations of **1**. Meanwhile, Oikawa and coworkers also proposed that a few compounds have mismatched stereochemistry. Therefore, it can explain that compound **2** possesses an °R_{ER}-methyl group at C-15 and an °S_{KR}-hydroxy group at C-17.

[1] Takino, T.; Kotani, A.; Ozaki, T.; Peng, W.Q.; Yu, J.; Guo, Y.; Mochizuki, S.; Akimitsu, K.; Hashimoto, M.; Ye, T.; Minami, A.; Oikawa, H. Biochemistry-guided prediction of the absolute configuration of fungal reduced polyketides. *Angew. Chem. Int. Ed.* **2021**, 60, 23403–23411.

Figure S32. MS/MS data of oxapyrrolidines (**1**-**2**) and asterisk-marked compounds.

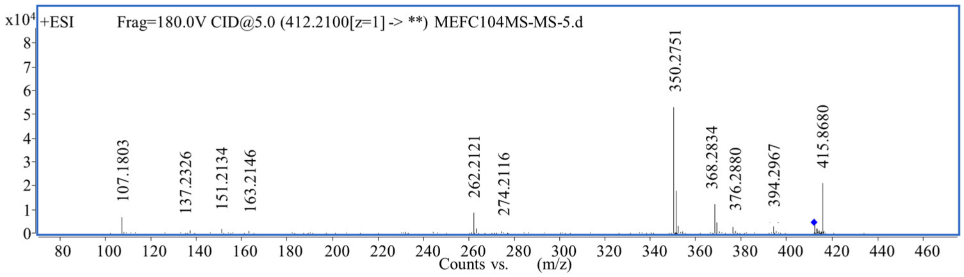

Oxapyrrolidines A (1)

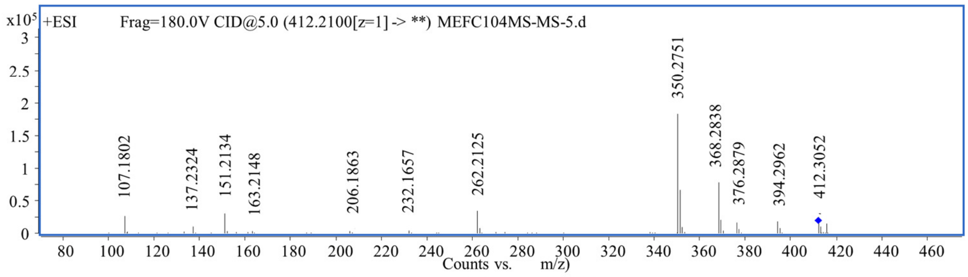

Oxapyrrolidines B (2)

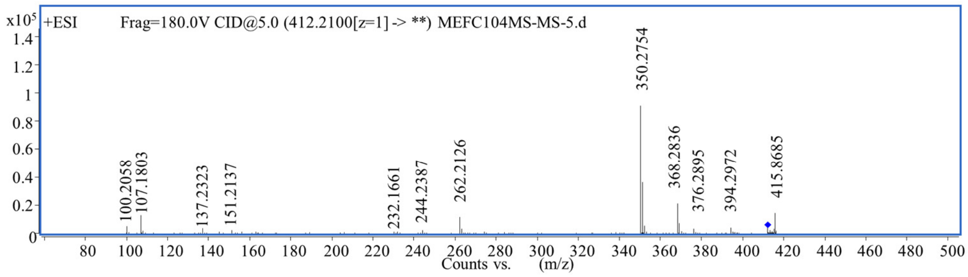

21.6 min (3)

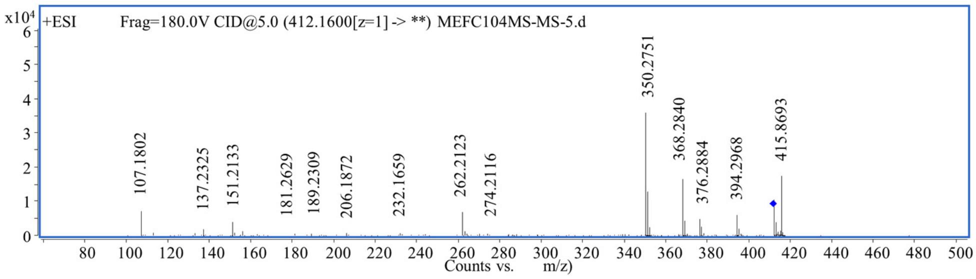

22.3 min (4)

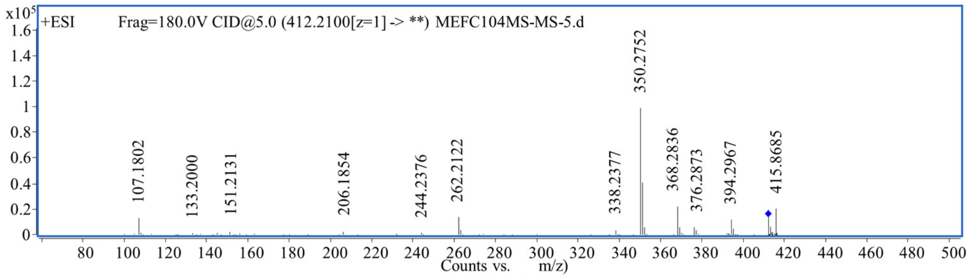

25.6 min (5)

Figure S33. Fragmentation pattern of oxapyrrolidines based on MS/MS data.

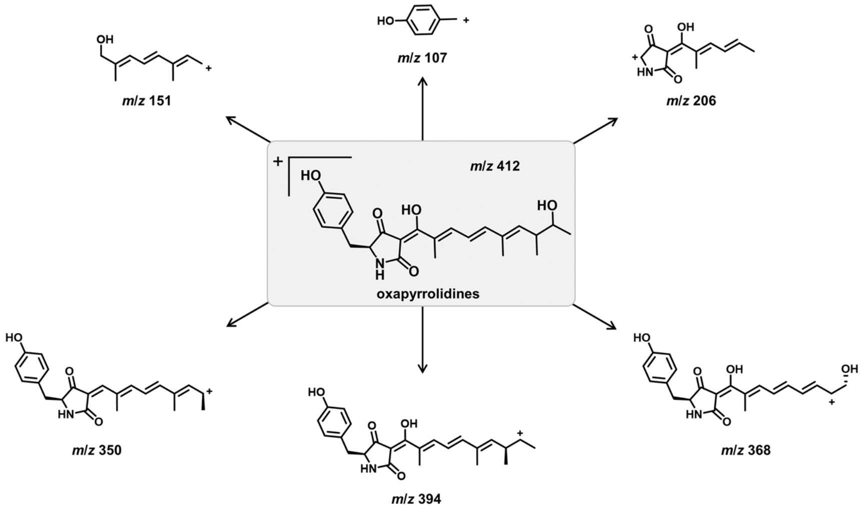

Compounds **1**-**2** and its analogs have the similar MS/MS patterns, such as the ion fragments of *m*/*z* = 394, 368, 350, 206, 151, 107 (Figure S32). We have assigned the structural fragments corresponding to daughter ions, including *p*-hydroxy benzyl, *α*-cleavage of the tetramic acid moiety, and the degradation of the aliphatic side chain. This information suggested that the structures of asterisk-marked in HPLC profiles were similar to that of **1**-**2**. The difference between **1**-**2** and its analogs might be the keto-enol tautomerization in tetramic acid moiety and *cis-trans* isomerization in the linkage of the aliphatic side chain and tetramic acid.
